# Deoxycholic acid induces gastric intestinal metaplasia by activating STAT3 signaling and disturbing gastric bile acids metabolism and microbiota

**DOI:** 10.1080/19490976.2022.2120744

**Published:** 2022-09-06

**Authors:** Duochen Jin, Keting Huang, Miao Xu, Hongjin Hua, Feng Ye, Jin Yan, Guoxin Zhang, Yun Wang

**Affiliations:** a Department of Gastroenterology, the First Affiliated Hospital of Nanjing Medical University, Nanjing China; b First Clinical Medical College, Nanjing Medical University, Nanjing, China; c Department of Pathology, The First Affiliated Hospital of Nanjing Medical University, Nanjing, China

**Keywords:** Deoxycholic acid, TGR5, STAT3, KLF5, gastric intestinal metaplasia, carcinogenesis, bile acids metabolic profiling, gastric microbiota

## Abstract

Intestinal metaplasia (IM) is the inevitable precancerous stage to develop intestinal-type gastric cancer (GC). Deoxycholic acid (DCA) is the main bile acid (BA) component of duodenogastric reflux and has shown an increased concentration during the transition from chronic gastritis to IM associated with continued STAT3 activation. However, the mechanisms underlying how DCA facilitates IM in the gastric epithelium need exploration. We evaluated IM and bile reflux in corpus tissues from 161 subjects undergoing GC screening. Cell survival and proliferation, proinflammatory cytokine expression and TGR5/STAT3/KLF5 axis activity were measured in normal human gastric cells, cancer cells, and organoid lines derived from C57BL/6, FVB/N and insulin-gastrin (INS-GAS) mice treated with DCA. The effects of DCA on IM development were determined in INS-GAS mice with long-term DCA supplementation, after which the gastric bacterial and BA metabolic profiles were measured by 16S rRNA gene sequencing and LC-MS. We revealed a BA-triggered TGR5/STAT3/KLF5 pathway in human gastric IM tissues. In gastric epithelial cells, DCA promoted proliferation and apoptotic resistance, upregulated proinflammatory cytokines and IM markers, and facilitated STAT3 phosphorylation, nuclear accumulation and DNA binding to the KLF5 promoter. DCA triggered STAT3 signaling and the downstream IM marker KLF5 in mouse gastric organoids *in vitro* and *in vivo*. In INS-GAS mice, DCA promoted the accumulation of serum total BAs and accelerated the stepwise development of gastric IM and dysplasia. DCA induced gastric environmental alterations involving abnormal BA metabolism and microbial dysbiosis, in which the *Gemmobacter* and *Lactobacillus* genera were specifically enriched. *Lactobacillus* genus enrichment was positively correlated with increased levels of GCA, CA, T-α-MCA, TCA and β-MCA in DCA-administrated INS-GAS mice. DCA promotes nuclear STAT3 phosphorylation, which mediates KLF5 upregulation associated with gastric inflammation and IM development. DCA disturbs the gastric microbiome and BA metabolism homeostasis during IM induction.

## Introduction

In both sexes, gastric cancer (GC) was the third leading cause of cancer death worldwide in 2018.^1^ Intestinal-type GC, which is the most common histological subtype of GC, occurs following Correa’s model of multistep precancerous stages: chronic gastritis, atrophy, gastric intestinal metaplasia (IM) and dysplasia.^2,3^ During this process, IM is considered an “irreversible point” in gastric carcinogenesis.^4,5^ A systematic literature review comprising a total of 21 studies found that IM individuals showed a pooled odds ratio of 3.58 (95% CI, 2.71–4.73) to develop GC compared to those without IM.^6^ Therefore, the exploration of the mechanism underlying IM is promising for the clinical prevention and treatment of gastric mucosal malignant transformation.

It is widely believed that *Helicobacter pylori* (*H. pylori* or Hp) eradication can reduce the risk of GC. However, the prevention effect of Hp eradication on IM in the antrum has not been determined, whereas it seems to have no preventive effect on IM in the corpus.^7–10^ Interestingly, several independent clinical studies have found that high concentrations of bile acids (BAs) in the stomach were associated with elevated risks of IM and GC in cases both with and without Hp infection.^11–14^ Deoxycholic acid (DCA) is one of the most hydrophobic secondary BAs in the human body^15^ and the main BA component of duodenogastric reflux (DGR, bile reflux).^16^ The concentration ratio of DCA to its precursor primary BA cholic acid in gastric juice samples showed an incremental increase during the progression of chronic superficial gastritis into IM and GC.^17^ G protein-coupled bile acid receptor 1 (GPBAR1, also called TGR5) is expressed in the gastric epithelium and activated by secondary BAs, especially DCA.^18^ Strong TGR5 staining was not present in normal gastric mucosa but was present in 12% of IM cases (*P* < .01).^19^ Therefore, the DCA-TGR5 axis may play a pivotal role in IM initiation.

Signal transducer and activator of transcription 3 (STAT3) is a transcription factor that is extensively involved in proinflammatory oncogenic cellular processes by regulating the expression of target genes in numerous solid tumors.^20,21^ Cytokines such as interleukin-6 (IL-6) phosphorylate the STAT3 protein on tyrosine-705 and subsequently induce the nuclear translocation of STAT3, which results in DNA binding and thereby transcriptional activation of STAT3 target genes.^22^ DCA activated STAT3 signaling and its transcriptional activity and thus promoted Barrett’s carcinogenesis.^23,24^ Wild-type mice showed more severe gastric inflammation and mucous metaplasia than gastric epithelial conditional *Stat3*-knockout mice.^25^ Hence, the elucidation of the mechanism underlying IM transformation from chronic gastritis based on DCA-TGR5-induced STAT3 activation is of great significance for increasing the comprehension of gastric mucosal carcinogenesis.

We aimed to fully investigate KLF5 expression during IM development in this study because substantial nuclear KLF5 staining was observed in 85.7% of patients with Barrett’s esophagus, and KLF5 responded positively to DCA-mediated intestinal transdifferentiation of the esophageal squamous epithelium.^26^ Moreover, the positive expression rate of KLF5 increased gradually, with significant differences in normal gastric tissues (38.5%), low-grade gastric intraepithelial neoplasia (GIN; 58.3%), high-grade GIN (66.7%), well-moderately differentiated adenocarcinoma (75.0%) and poorly differentiated adenocarcinoma (78.9%).^27^

Transgenic FVB/N insulin-gastrin (INS-GAS) mice initially showed an increased parietal cell number but later exhibited parietal cell loss and hypochlorhydria, which could spontaneously progress to gastric IM, dysplasia and cancer at 20 months of age, and these mice are considered to be an ideal animal model for GC research.^28^ In addition to Hp, gastric microbial dysbiosis has been determined to be involved in mucosal carcinogenesis in the stomach.^29,30^ The colonization densities of the bacterial community in the stomach are estimated to range from 10^1^ to 10^3^ colony forming units/g, and an altered community structure along with decreased microbial diversity might promote GC.^31,32^ Hp-negative individuals can have better phylotype evenness and diversity on the composition of the gastric microflora than subjects positive for *H. pylori*,^33^ and Hp-infected INS-GAS mice with more diversified gastric commensal bacteria were observed to have less severe gastric lesions and delayed GC onset compared to Hp monoinfected mice.^34^ It is consequently necessary to explore the effects of DCA on BA metabolism and the microbial diversity and community structure in the stomachs of INS-GAS mice to validate potential microbial markers for IM occurrence and progression.

In this study, we aimed to explore the involvement of TGR5-STAT3-KLF5 signaling axis on gastric IM development in response to DCA and the effects of DCA treatment on the BA metabolism and gastric microbiota of INS-GAS mice. Therefore, we investigated the roles of STAT3 signaling in KLF5 activation in response to BAs in the gastric epithelium. We found that DCA might promote the transformation of gastric mucosal inflammation to IM and carcinogenesis. The involvement of a TGR5/STAT3/KLF5 regulatory axis was observed in human IM tissues exposed to regurgitant bile, in the gastric organoids of mice treated with DCA, and in DCA-fed INS-GAS mice. Furthermore, long-term DCA supplementation in drinking water also induced gastric BA metabolism and microbiota dysbiosis during IM and dysplasia onset. These findings reveal a critical role of STAT3 signaling in regulating its target intestinal marker KLF5 in response to DCA, which is the main BA component in the stomach and the major risk factor for gastric IM.

## Results

### Bile reflux caused increased high levels of TGR5, p-STAT3 and KLF5 expression in the human gastric epithelium

**Figure 1.**
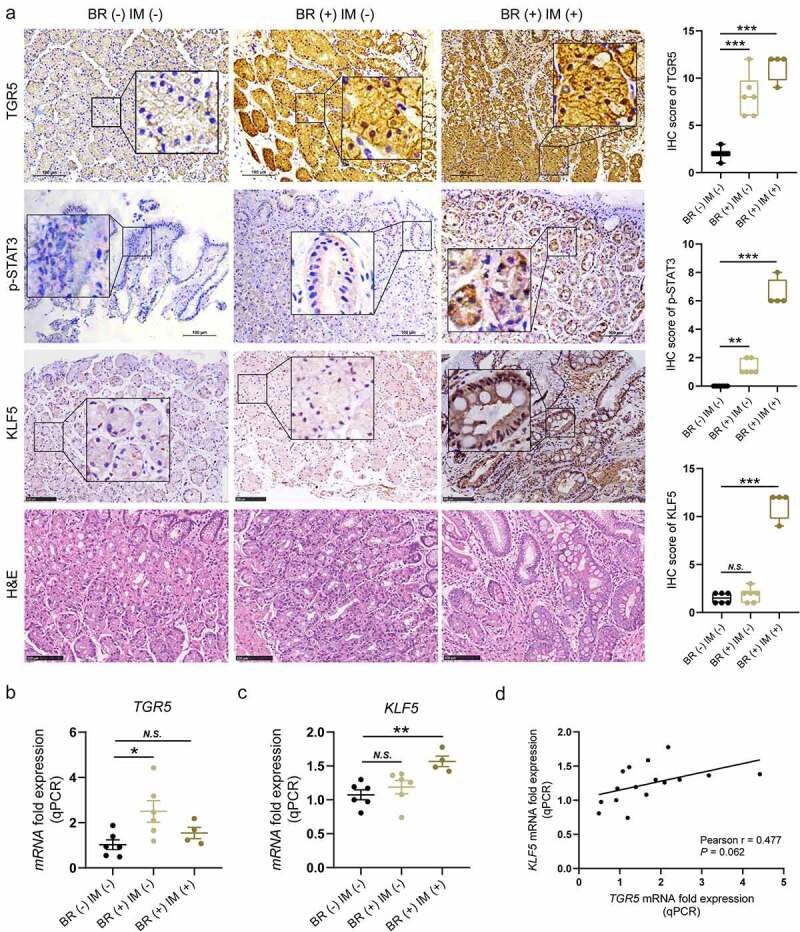
Enhanced TGR5, p-STAT3 and KLF5 expression was observed in the human gastric epithelium in patients with bile acid reflux. (a) IHC staining and scoring of TGR5, p-STAT3 and KLF5 in the gastric mucosal biopsy specimens of the enrolled 16 subjects. (b-c) mRNA expression levels of TGR5 and KLF5 in gastric mucosal samples from the 16 included individuals, as assessed by qRT-PCR assays. (d) Pearson correlation analysis of KLF5 and TGR5 mRNA expression levels. Scale bar, 100 μm. *P < .05, **P < .01, ***P < .001. *N.S*., not significant.

Sixteen subjects were enrolled from a provincial GC early detection project that screened a total of 161 volunteers without Hp infection in 2019, including 6 presenting BR (-) IM (-), 6 showing BR (+) IM (-), and 4 with BR (+) IM (+). BR represents bile reflux. IHC staining and scoring of TGR5, p-STAT3 and Kruppel-like Factor 5 (KLF5) expression levels in the gastric biopsy samples were performed for the 16 enrolled individuals (Figure 1a). As shown in Figure 1a and Table 1, membranal TGR5 staining and nuclear p-STAT3 staining of the gastric epithelium were both stepwise and statistically enhanced in BR (-) IM (-), BR (+) IM (-) and BR (+) IM (+) biopsies. Although nuclear KLF5 staining in BR (+) IM (-) tissues did not show a significant increase compared with that in BR (-) IM (-) tissues (-, 1.50 ± 0.22 *vs*. -, 1.83 ± 0.31; *P* > .05), the substantial nuclear KLF5 staining in BR (+) IM (+) tissues was significantly different from that in BR (-) IM (-) tissues (-, 1.50 ± 0.22 *vs*. ++, 11.25 ± 0.75; *P* < .001). The TGR5 and KLF5 mRNA expression levels further detected by qRT-PCR assays were similar to their protein expression trends determined based on IHC staining (Figure 1b-c). Interestingly, the KLF5 mRNA level had a moderate positive correlation with the TGR5 mRNA level in the gastric tissues of the 16 included subjects (r = 0.477, *P* = .062; Figure 1d).

**Table 1.** Expressions of TGR5, p-STAT3 and KLF5 in BR (-) IM (-), BR (+) IM (-) and BR (+) IM (+) human gastric epithelia based on IHC staining.

Target name	Score, mean ± SEM			*P*_1_ value	*P*_2_ value
	BR (-) IM (-)	BR (+) IM (-)	BR (+) IM (+)		
TGR5	-, 2.00 ± 0.26	++, 8.17 ± 0.91	++, 11.25 ± 0.75	< 0.001	< 0.001
p-STAT3	-, 0	-, 1.33 ± 0.21	+, 6.50 ± 0.50	< 0.01	< 0.001
KLF5	-, 1.50 ± 0.22	-, 1.83 ± 0.31	++, 11.25 ± 0.75	> 0.05	< 0.001

^1^BR (-) IM (-) group *vs*. BR (+) IM (-) group with an unpaired parametric *t* test. ^2^ BR (-) IM (-) group *vs*. BR (+) IM (+) group with an unpaired parametric *t* test. IHC, immunochemistry; -, negative staining; +, weak staining; ++, strong staining.

### DCA showed cytotoxicity, promoted proliferation and apoptotic resistance, and could activate STAT3 phosphorylation in gastric cells

**Figure 2.**
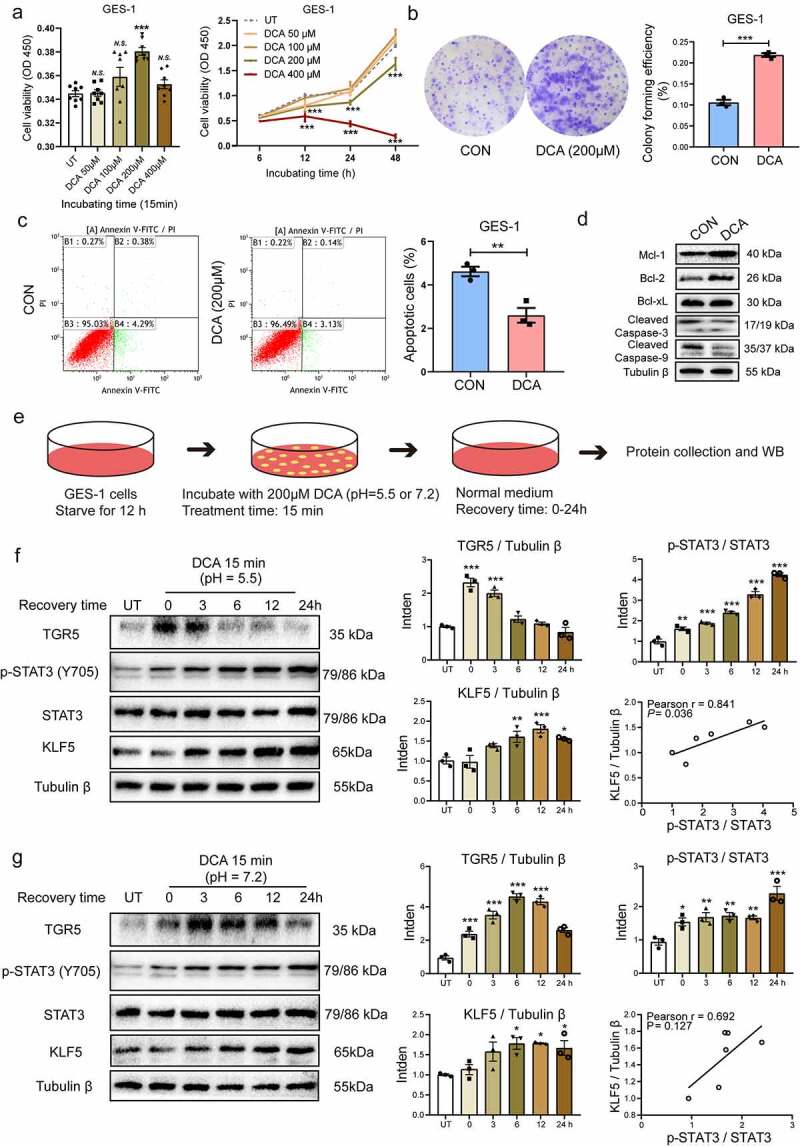
DCA induced cytotoxicity, promoted proliferation, inhibited apoptosis, and activated STAT3 phosphorylation in gastric cells. (a) CCK-8 assays were used to examine cell proliferation/cytotoxicity in GES-1 cells treated with DCA at different doses for different times. (b) Short-term stimulation with 200 µM DCA for 15 min significantly promoted colony formation in GES-1 cells. (c) Apoptosis rates in GES-1 cells exposed to 200 µM DCA for 15 min and subsequently incubated in normal medium for 24 h, as measured by flow cytometry. (d) Protein expression levels of antiapoptotic Mcl-1, Bcl-2 and Bcl-xL and apoptotic cleaved caspase-3 and −9 in GES-1 cells under the same DCA exposure conditions examined by western blotting. (e) Workflow used to examine the different effects of neutral and acidic DCA (pH = 7.2 and 5.5) on inflammation and IM phenotypes in GES-1 cells. (f-g) Western blotting to determine the protein levels of TGR5, phosphorylated STAT3 and KLF5 in GES-1 cells treated with DCA under acidic and neutral conditions. The data are represented as the mean ± SEM. *P < .05, **P < .01, ***P < .001.

**Figure 3.**
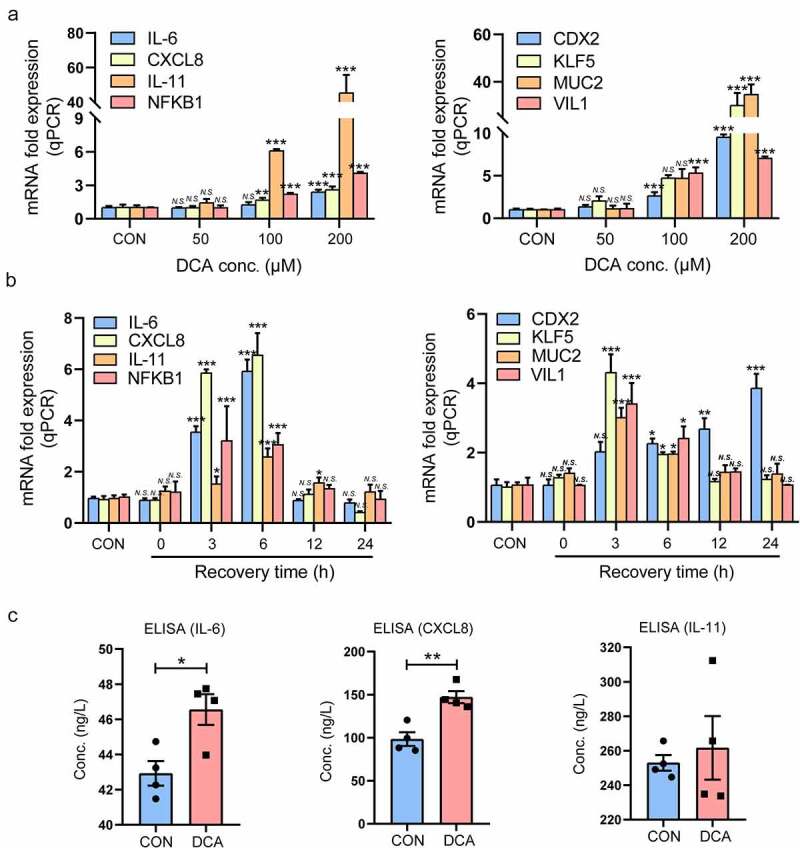
DCA upregulated proinflammatory cytokines and intestinal markers in gastric cells. (a) DCA dosages: 50, 100 and 200 µM in 24 h interventions. The mRNA expression levels of IL-6, CXCL8, IL-11, NFKB1, CDX2, KLF5, MUC2 and VIL1 were then detected by qRT-PCR assays. (b) DCA dosage: 200 µM in a 15 min intervention; recovery times: 0, 3, 6, 12, and 24 h. The mRNA expression levels of the abovementioned genes were then detected with qRT-PCR. (c) DCA dosage: 200 µM in a 15 min intervention; recovery time: 24 h. The concentrations of IL-6, CXCL8 and IL-11 in the culture medium of GES-1 cells after DCA stimulation were determined by ELISA. *P < .05, **P < .01, ***P < .001. *N.S*., not significant.

Given that DCA is the main BAs component in DGR,^16^ we wanted to examine the phenotypic changes in immortalized GES-1 gastric epithelial cells treated with DCA. CCK-8 assays were used to detect the viability-inhibitory effects in GES-1 cells exposed to different concentrations of DCA (50–400 μM) for 15 min, 6, 12, 24, and 48 h. It was found that 200 μM DCA significantly enhanced GES-1 cells viability after stimulation for only 15 min (*P* < .001). Low concentrations of DCA (50 or 100 μM) did not inhibit cell viability compared with the untreated control at each time point. However, cell viability was significantly decreased by 81.7% after 48 h of treatment with 200 μM DCA, while 400 μM DCA showed marked cytotoxicity (Figure 2a). Hence, continuous DCA intervention showed dose-dependent cytotoxicity to GES-1 cells. Based on our subsequent experiments, DCA upregulated proinflammatory cytokines and intestinal markers and induced TGR5 expression and simultaneous STAT3 phosphorylation in a dose-dependent manner, with the greatest effects at 200 μM (Figure 3a and **Fig. S1E**). Therefore, 200 μM DCA was chosen for further exploration. Colony formation assays were conducted to observe the proliferative capacity of GES-1 cells that received short-term stimulation with 200 μM DCA for 15 min. DCA was used in this study for a short time span (15 min) to mimic reflux episodes. The results indicated that DCA significantly promoted the number and size of colonies formed by gastric cells (*P* < .001; Figure 2b). Afterward, the apoptosis rate of GES-1 cells exposed to 200 μM DCA for 15 min and subsequently incubated in normal medium for 24 h of recovery was measured by flow cytometry. The results showed that the number of apoptotic cells in the DCA group was remarkably lower than that in the control group (*P* < .01; Figure 2c), suggesting that DCA might induce apoptotic resistance in gastric cells. To confirm these findings, we further performed western blotting to detect the expression levels of a series of apoptotic and antiapoptotic proteins in GES-1 cells under the same DCA exposure conditions. The results revealed that DCA induced resistance to apoptosis through upregulation of the protein expression levels of anti-apoptotic Mcl-1 and Bcl-2 and downregulation of the protein expression levels of apoptotic cleaved caspase-3 and caspase-9 in GES-1 cells (Figure 2d and **Fig. S4A**). The findings demonstrated that DCA had cytotoxic effects on gastric epithelial cells, but the viable cells tended to resist apoptosis under DCA stimulation.

Since acidic bile reflux clearly contributes to the development of Barrett’s esophagus and its progression to adenocarcinoma,^35,36^ at the end of this experiment, we aimed to clarify the different effects of neutral and acidic DCA on inflammation and IM phenotypes in normal human gastric epithelial cells. Briefly, GES-1 cells were treated with 200 μM DCA in neutral and acidified media (pH = 7.2 and 5.5, respectively) for 15 min and then returned to normal medium for different times (0–24 h; Figure 2e). The protein expression levels of the DCA receptor TGR5, phosphorylated STAT3 and its potential target gene KLF5, an IM marker, were detected by western blotting (figure 2f-g). The results showed that the expression of TGR5 was increased 15 minutes after the stimulation of GES-1 cells with either acidic DCA or neutral DCA, and neutral DCA had a more lasting activation effect, which lasted up to 12 hours after DCA withdrawal. Moreover, STAT3 phosphorylation (Tyr705) and KLF5 expression were significantly increased with the extension of the recovery time after acidic or neutral DCA treatment. Interestingly, there were positive correlations between p-STAT3 and KLF5 protein expression in both the acidic and neutral DCA treatment groups (Pearson’s r = 0.841, *P* = .036; Pearson’s r = 0.692, *P* = .127). Therefore, we observed that DCA promoted gastric epithelial inflammation and IM, regardless of the pH value, which is similar to the previously reported roles of DCA in esophageal mucosa.^36,37^ Given that the loss of parietal cells in the gastric body results in diminished gastric acid secretion and elevated gastric juice pH in atrophic gastritis and IM,^38^ neutral DCA was selected for subsequent experiments studying IM development in the gastric body.

### DCA upregulated proinflammatory cytokines and intestinal markers in gastric epithelial cells

To observe the significance of DCA in boosting gastric inflammation and IM, immortalized GES-1 gastric epithelial cells were treated with DCA at different doses for different times. The mRNA expression levels of proinflammatory cytokines and intestinal markers were determined by qRT-PCR after stimulation with DCA, and the protein concentrations of proinflammatory cytokines in the supernatant were detected by ELISA. Consequently, the mRNA expression levels of IL-6, CXCL8, IL-11, NFKB1, CDX2, KLF5, MUC2 and VIL1 were dose-dependently increased under DCA stimulation, and 200 μM DCA had the strongest effects (Figure 3a).

Next, GES-1 cells were treated with 200 μM DCA for 15 min and subsequently incubated in normal medium for different periods, and the results showed that the promotion effects of DCA on the mRNA expression of these genes lasted for 3–6 hours after DCA withdrawal (Figure 3b). In addition, since IL-6, CXCL8 and IL-11 are secretory proteins that exert initial proinflammatory signal effects, we also detected the concentrations of these proinflammatory cytokines in the cell culture medium using ELISA. The results showed that the IL-6 and CXCL-8 concentrations were significantly higher in the supernatant of GES-1 cells that received DCA stimulation than in the untreated cells (42.93 ± 0.70 ng/L *vs*. 46.56 ± 0.87 ng/L, *P* < .05; 98.34 ± 8.01 ng/L *vs*. 147.1 ± 7.03 ng/L, *P* < .01), and there was no difference in the IL-11 concentration between the two groups (Figure 3c).

### DCA-TGR5 axis facilitates STAT3 phosphorylation, nuclear accumulation, and transcriptional activation

TGR5 is a predominant G-protein-coupled receptor mediated by secondary BAs.^39^ Herein, we aimed to explore the role of DCA in regulating TGR5 expression. We initially examined the baseline expression of TGR5 mRNA and protein in the gastric epithelial cell line GES-1 and GC cell lines AGS, SGC-7901, BGC-823 and MKN-45 using qRT-PCR and western blotting. As shown in **Fig. S1A**, TGR5 mRNA and protein expression levels were lower in GES-1 cells than in the GC cells. Moreover, TGR5 mRNA expression levels were lower in AGS and SGC-7901 cells than in BGC-823 and MKN-45 cells. Therefore, we selected a low-expressing cell line AGS and a high-expressing cell line BGC-823, respectively, for subsequent DCA intervention experiments. Next, GES-1 cells were treated with 200 μM DCA for 15 min and incubated in normal medium for different times. The qRT-PCR results revealed that the expression of TGR5 mRNA reached the highest level at 3 h of recovery culture and then gradually returned to the baseline level (**Fig. S1B**). The immunofluorescence results also showed increased expression of TGR5 in the cytoplasm and membrane of GES-1 cells and AGS and BGC-823 GC cells stimulated by DCA with a 3-hour recovery (**Fig. S1C-D**). Furthermore, we treated GES-1 cells with DCA at different doses with a 3-hour recovery culture. The qRT-PCR and western blotting results showed that compared with untreated cells, DCA induced TGR5 expression and simultaneous STAT3 phosphorylation in a dose-dependent manner, with the greatest effects at 200 μM (**Fig. S1E**). Based on these results together, DCA mediated the expression of the secondary BA receptor TGR5 on the membrane of gastric epithelial cells.

**Figure 4.**
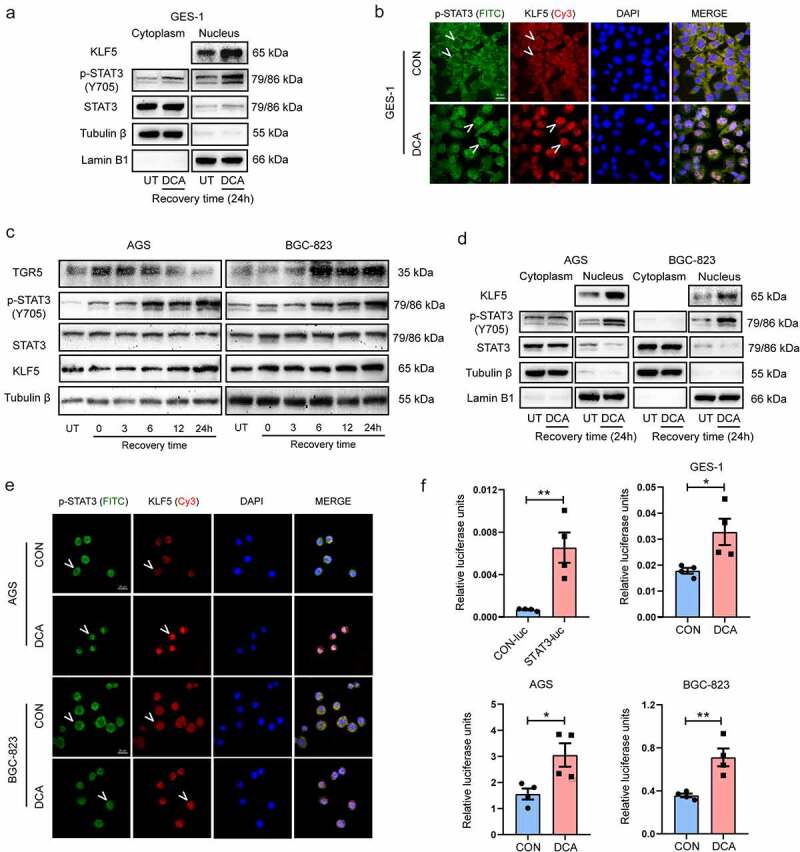
DCA-TGR5 axis facilitated STAT3 phosphorylation, nuclear accumulation and transcriptional activation. After 15 min of exposure to 200 μM DCA followed by 24 h of recovery in fresh complete media, the cytoplasmic and nuclear proteins in GES-1 cells (a) and AGS and BGC-823 cells (d) were completely separated and then detected by western blotting. Immunofluorescence images of GES-1 cells (b) and AGS and BGC-823 cells (e) treated with and without DCA under the same conditions mentioned above. Scale bars, 20 μm. (c) Gastric cancer cells AGS and BGC-823 were treated with 200 μM DCA for 15 min, followed by different recovery times in complete media. TGR5, p-STAT3 and KLF5 expression in the total cell fractions was detected by immunoblot analysis. (f) Luciferase reporter assays examining STAT3 transcriptional activity were performed in GES-1, AGS and BGC-823 cells after transfection with the p-Stat3-Luciferase reporter followed by DCA treatment (200 µM) for 15 min. The reporter activity was measured post recovery in complete media for 24 h. UT, untreated. *P < .05, **P < .01.

STAT3 is a transcription factor involved in gastric tumorigenesis. Detection based on clinical specimens found that tyrosine-phosphorylated STAT3 (p-STAT3) expression was elevated in GC and precancerous lesions, including IM and dysplasia,^40,41^ the underlying molecular mechanism of which requires further investigation. We first investigated the phosphorylation and localization of STAT3 in DCA-treated GES-1 cells. Transient stimulation with DCA mimicking a reflux episode resulted in remarkable increases in p-STAT3 and KLF5 levels in the nuclear fraction (Figure 4a and **Fig. S4B**). We further observed that DCA treatment drove the nuclear accumulation of p-STAT3 and KLF5 in GES-1 cells compared to untreated cells (Figure 4b). Similar to the effects in gastric normal epithelial cells, in two gastric cancer cell lines, AGS and BGC-823, the secondary bile acid receptor TGR5 was significantly activated after transient DCA stimulation, the expression of p-STAT3 and KLF5 increased stepwise with prolonged recovery time (0–24 h), and activated p-STAT3 mainly accumulated in the nucleus (Figure 4c-e). To investigate whether DCA-mediated nuclear p-STAT3 accumulation influenced STAT3 transcriptional activity, the validity of the p-Stat3-Luciferase reporter was first confirmed. The reporter was successfully transfected into GES-1 cells and indeed enhanced the luciferase signal (*P* < .01). Then, on this basis, a significant increase in STAT3 transcriptional activity was found in normal and cancerous gastric cells after DCA stimulation (figure 4f). Taken together, the DCA-TGR5 axis facilitated nuclear p-STAT3 accumulation and STAT3 transcriptional activation, which may be related to KLF5 overexpression.

### After DCA treatment, p-STAT3 activates KLF5 expression by binding to the KLF5 promoter in gastric cells

**Figure 5.**
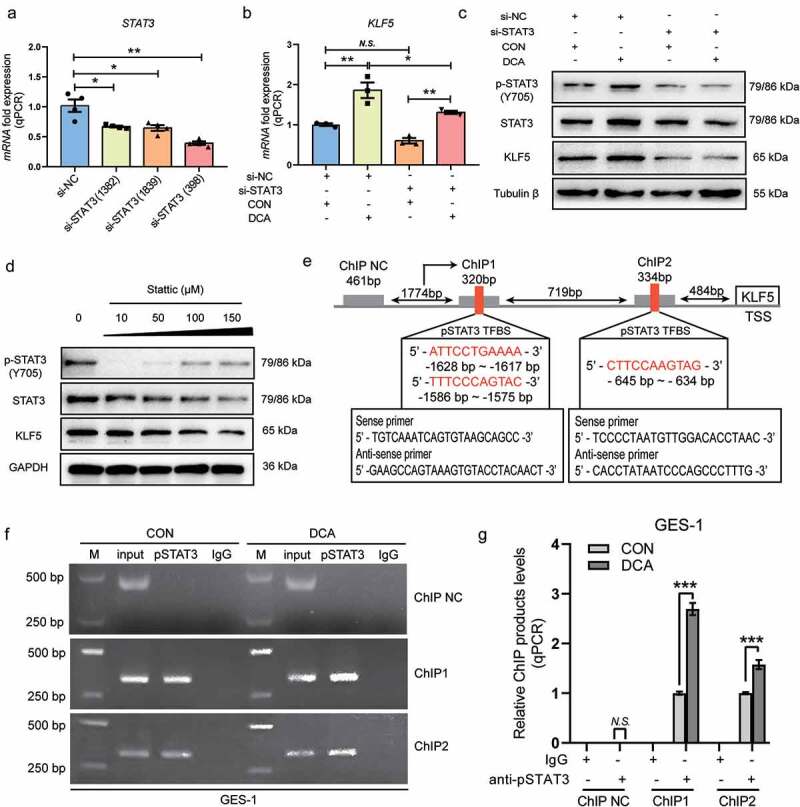
After DCA treatment, p-STAT3 activates KLF5 expression by binding to the KLF5 promoter in gastric cells. (a) STAT3 mRNA levels detected by qRT-PCR in GES-1 cells 24 h after si-STAT3 transfection with different oligonucleotide sequences. (b-c) GES-1 cells were transfected with 30 µM si-STAT3 (398) for 24 h and stimulated with 200 µM DCA for 15 min followed by 24 h of recovery. KLF5 mRNA expression was detected by qRT-PCR, and p-STAT3, STAT3 and KLF5 protein expression was examined by western blotting. (d) After the inhibition of STAT3 phosphorylation by Stattic (Cons. 10–150 µM) for 2 hours, as recommended in the manufacturer’s instructions, KLF5 protein expression was detected by western blotting. (e) Schematic representation of the KLF5 promoter, in which two putative STAT3 binding sites and the primers used for ChIP assays are shown. (f) p-STAT3 bound to the KLF5 promoter in untreated GES-1 cells and GES-1 cells treated with 200 µM of DCA for 15 min with subsequent recovery in normal medium for 24 h was detected by ChIP assays. (g) Enrichment levels of p-STAT3 binding to the KLF5 promoter were significantly increased according to q-PCR detection with each of the two primers. *P < .05, **P < .01, ***P < .001. *N.S*., not significant.

To confirm that KLF5 expression is regulated by STAT3, especially the active form of STAT3 (p-STAT3), we first silenced STAT3 using siRNAs in GES-1 cells. It was observed that si-STAT3 transfection with each of the three oligonucleotide sequences could significantly inhibit STAT3 mRNA expression (Figure 5a), and we selected si-STAT3, which had the strongest inhibitory effect, for use in the following experiment. In cells not treated with DCA, si-STAT3 transfection did not change the KLF5 mRNA levels. In cells that received 200 μM DCA stimulation for 15 min, pretransfection with si-STAT3 resulted in a significant decrease in KLF5 mRNA levels (Figure 5b). The inhibition of KLF5 at the protein level by si-STAT3 transfection was also analyzed, and equivalent results were obtained (Figure 5c and **Fig. S4C**). To further assess the implication of STAT3 phosphorylation in regulating KLF5 expression, GES-1 cells were treated with a selective STAT3 inhibitor, Stattic, which inhibits the phosphorylation, dimerization and nuclear translocation of STAT3 by interacting with the SH2 domain. The addition of Stattic dose-dependently decreased the expression of KLF5 (Figure 5d and **Fig. S4D**).

*In vivo* binding of the active form of STAT3 (p-STAT3) to the KLF5 promoter was further confirmed by ChIP assays after two putative binding sites for STAT3 were located within 2 kb of the KLF5 proximal promoter through the JASPAR website (https://jaspar.genereg.net/; Figure 5e). Two specific primers, one including two STAT3-binding sites in the KLF5 promoter used for the ChIP experiments, were designed and are shown in Figure 5e. Consequently, no binding was detected when a negative control primer pair was used for q-PCR, whereas visible binding of p-STAT3 to the KLF5 promoter was observed in untreated GES-1 cells. Furthermore, a significant increase in binding was detected in GES-1 cells treated with DCA when using each of the primers (figure 5fandg). Together, DCA might promote STAT3 phosphorylation and subsequently activate KLF5 transcription through the direct binding of p-STAT3 to the KLF5 promoter.

### DCA activates STAT3 signaling and the downstream IM marker KLF5 in mouse gastric organoids *in* *vitro* and *in* *vivo*

**Figure 6.**
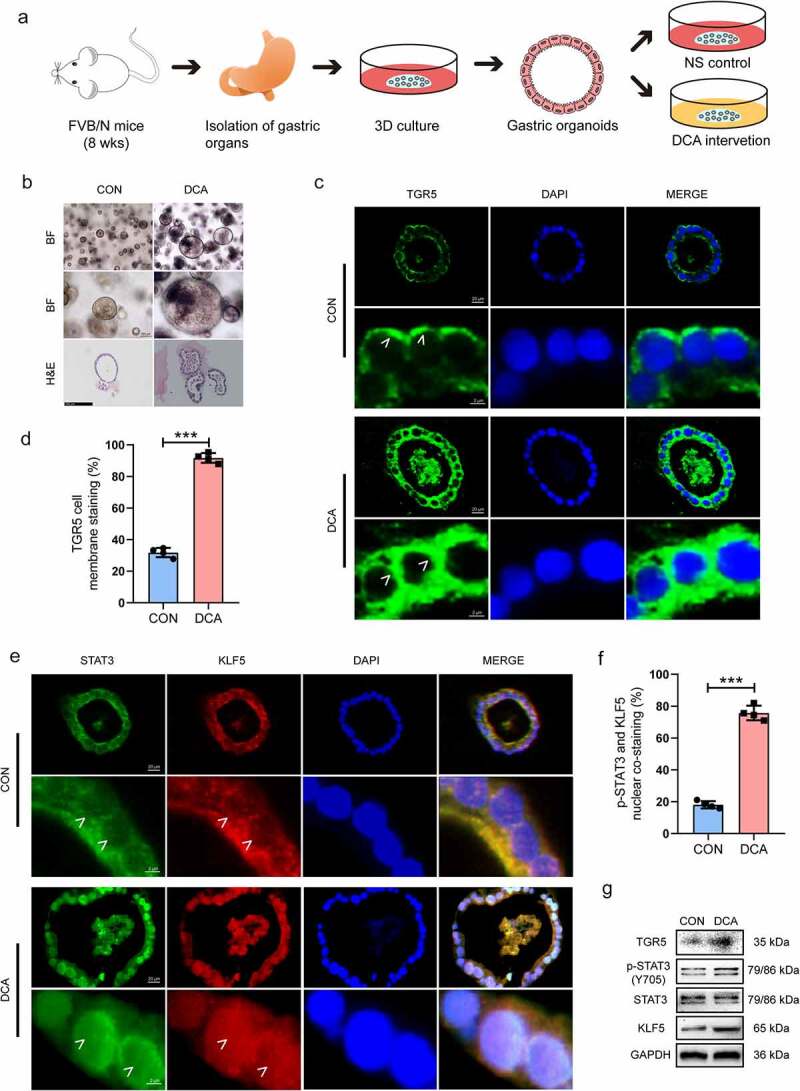
DCA promoted STAT3 phosphorylation and the expression of the IM marker KLF5 in gastric organoids derived from FVB/N mice *in vitro*. (a) Workflow used to examine the phenotypic changes in gastric organoids treated with DCA *in vitro*. Dosage: 200 µM for 15 min; recovery time: 24 h in normal medium. (b) Bright-field microscopy and hematoxylin and eosin staining of representative stomach organoids of FVB/N mice treated with NS or DCA. Scale bars, 200 μm (top) and 100 μm (middle and bottom). (c-f) Immunofluorescence staining for TGR5, p-STAT3 and KLF5 in gastric organoids from FVB/N mice stimulated with DCA *in vitro*. Scale bars, 20 μm (top) and 2 μm (bottom). (g) Western blot analysis of gastric organoids from FVB/N mice stimulated with DCA *in vitro*. NS, normal saline. ***P < .001.

Given that gastric organoids better mimic the genetic, phenotypic and behavioral traits of normal stomach tissues and tumors than frequently used cell lines,^42^ we further conducted *in vitro* and *in vivo* mouse organoid experiments to explore the regulatory effects of DCA on the TGR5/p-STAT3/KLF5 axis of the gastric epithelium. The workflow used to examine the phenotypic changes in gastric organoids treated with DCA *in vitro* is shown in Figure 6a. Stomach organoids were extracted from three 8-week-old male FVB/N mice and treated with DCA *in vitro*. Representative bright-field microscopic and H&E-stained images of the stomach organoids in the two groups are shown in Figure 6b. Immunofluorescence staining analysis showed that the basal levels of TGR5 membrane staining (Figure 6c-d) and p-STAT3 and KLF5 nuclear costaining (Figure 6e-f) were low in gastric organoids, and these levels could be increased by DCA *in vitro* [(31.80 ± 1.48)% *vs*. (91.70 ± 1.55)%, *P* < .001; (18.11 ± 1.14)% *vs*. (75.74 ± 2.30)%, both *P* < .001]. Western blot analysis was also performed, and a similar trend to that shown in the IF assays was observed (Figure 6g and **Fig. S4E**).

**Figure 7.**
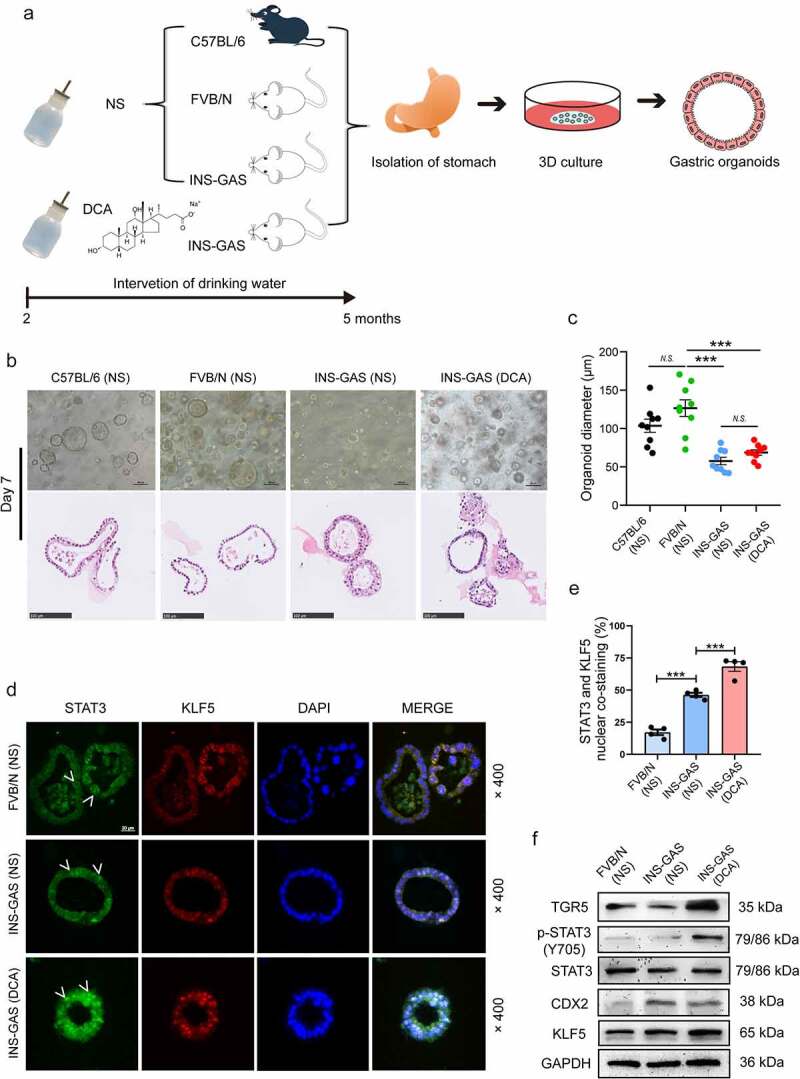
STAT3 phosphorylation and the IM phenotype were observed in gastric organoids derived from INS-GAS transgenic mice after drinking DCA for 3 months. (a) Schematic diagram of the *in vivo* experimental procedure. (b) Stomach organoids derived from the four groups of mice were extracted and cultured for 7 days for bright-field microscopy examination and H&E staining. Scale bar, 100 μm. (c) For the statistical analyses of the average maximum diameters of the four groups of organoids, three microscopic fields (under 100× magnification) were randomly selected from each sample, and the three largest organoids in each field were selected for diameter measurement. (d) Immunofluorescence staining for nuclear p-STAT3 and KLF5 coexpression in gastric organoids from FVB/N (NS), INS-GAS (NS) and INS-GAS (DCA) mice. Scale bar, 20 μm. (e) Quantification of nuclear p-STAT3 and KLF5 costaining in the three groups. (f) Western blot analysis of gastric organoids from FVB/N (NS), INS-GAS (NS) and INS-GAS (DCA) mice. ***P < .001; *N.S*., not significant.

We next verified our findings regarding DCA-mediated gastric IM using three mouse stomach organoid lines *in vivo*. As shown in Figure 7a, a total of three C57BL/6 mice, three FVB/N wild-type mice and six FVB/N INS-GAS mice were prepared, all of which were male mice aged 8 weeks. Among them, three INS-GAS transgenic mice were randomly selected to be given free access to 0.2% DCA drinking water, as previously reported,^43^ and all remaining mice were given NS in their drinking water. In this part of the *in vivo* experiments, we used NS as the solvent for DCA instead of DMEM to eliminate the bias caused by the toxicity of DMEM. Three months after the drinking water intervention, gastric organoids were extracted and cultured for 7 days, and then the sizes of the organoids in the four groups were observed by bright-field microscopy and H&E staining (Figure 7b). A previous study found that INS-GAS mice showed decreased maximal gastric acid secretion and decreased parietal cell number in later stage (five months and older),^28^ which suggests the beginning of gastric atrophy. Hence, we selected the three months of DCA administration for the organoids *in vivo* experiments. The average maximum diameter of the organoids was 103.64 μm in the C57BL/6 mouse group, 126.57 μm in the FVB/N mouse group, 57.55 μm in the INS-GAS mouse group, and 68.78 μm in the INS-GAS (DCA) mouse group. Compared with FVB/N wild-type mice, INS-GAS transgenic mice showed significant glandular atrophy regardless of whether they received DCA (both *P* < .001; Figure 7c). Immunofluorescence staining images and the results of quantification of the nuclear coexpression of p-STAT3 and KLF5 are shown in Figure 7d **and** e, indicating obvious enhancement in organoids extracted from INS-GAS mice that received DCA administration compared to those from untreated INS-GAS mice (*P* < .001). figure 7f and **Fig. S4F** show the western blot results, which were consistent with the immunofluorescence photomicrographs. Moreover, DCA ingestion did not affect gastric mucosal CDX2 expression, although it was more highly expressed in INS-GAS mice.

We also collected and mixed the gastric contents of each group of mice (*n* = 3) to prepare gastric content supernatants used to stimulate GES-1 cells, aiming to observe the effects of long-term DCA ingestion by INS-GAS mice on the TGR5/STAT3/KLF5 axis of normal human gastric epithelial cells. Promisingly, the results showed that the gastric extracts of the INS-GAS (DCA)-group mice also promoted inflammation and IM transformation in GES-1 cells (**Fig. S2**). Therefore, in the next experiment, we further explored the effects of DCA on the gastric environment of INS-GAS mice from the perspectives of gastric bile acid metabolism profiling and gastric bacterial alteration. Taken together, the data from the gastric epithelial cell and organoid experiments indicate that DCA might play a vital role in multistep precancerous processes involving inflammation and IM and the transformation from chronic inflammation to IM.

### DCA promotes the accumulation of serum TBA and accelerates the stepwise development of gastric IM and dysplasia in INS-GAS mice

**Figure 8.**
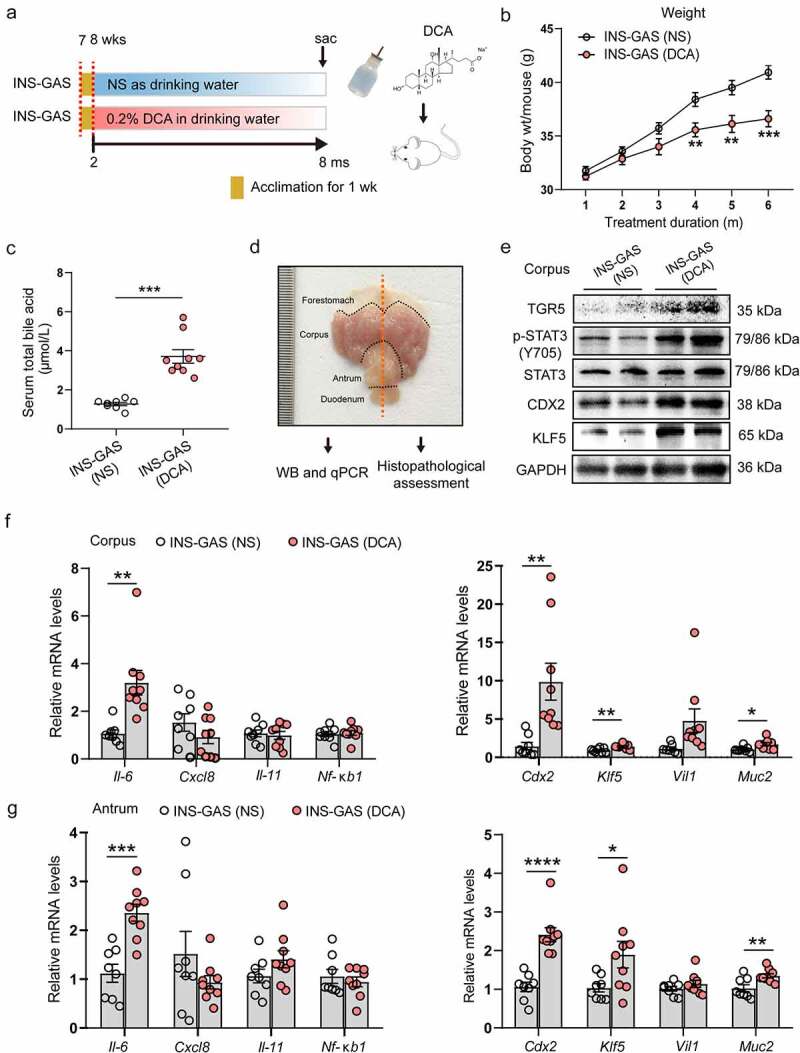
DCA resulted in the accumulation of serum TBA and enhanced STAT3 phosphorylation and IM marker expression in the gastric mucosae of INS-GAS mice. (a) Treatment schemata used for 0.2% DCA administration in the drinking water of INS-GAS mice. n = 8 INS-GAS (NS), n = 9 INS-GAS (DCA). (b) Body weight changes in the two groups of INS-GAS mice during the intervention period. (c) Concentrations of serum TBA in the two groups determined by the enzymatic cycling method. (d) Macroscopic-level schematic drawing of mouse stomach anatomy depicting the sampling sites used in each experiment. (e) Western blotting for TGR5, p-STAT3 and IM markers CDX2 and KLF5 in the corpus tissues of the two groups. (f and g) mRNA expression detection by qRT-PCR in the corpus and antrum tissues of the two groups of INS-GAS mice.

To understand the pathogenesis of gastric IM induced by BAs, we utilized a model of intestinal-type GC called transgenic INS-GAS mice, as previously reported.^28^ DCA in drinking water was given to mice aged 2 months, and the treatment was maintained for 6 months (Figure 8a). We found that beginning in the fourth month, DCA induced a significant body weight decrease in INS-GAS mice (Figure 8b). We further detected the serum TBA concentration in the two groups by the enzymatic cycling method and observed a remarkable accumulation of serum TBA in the DCA group compared to the NS group (1.28 ± 0.09 *vs*. 3.71 ± 0.35 μmol/L, *P* < .001; Figure 8c). For the subsequent analysis, a macroscopic-level schematic drawing of mouse stomach anatomy is shown in Figure 8d, which depicts the sampling sites used in each experiment. More specifically, after incision along the greater curvature of the stomach, the left portion of the stomach was used for western blotting and qPCR assays, while the right portion was used for histopathological assessments. As shown in Figure 8e and **Fig. S4G**, DCA treatment led to markedly increased expression of TGR5 and the IM markers CDX2 and KLF5 and to the phosphorylation of STAT3 in corpus tissues compared to the slight expression detected in NS-treated samples. We also observed significantly higher levels of IL-6, CDX2, KLF5 and MUC2 mRNA expression in both corpus and antrum tissues in the DCA group than in the control group (figure 8f,g), which were indicative of activated mucosal chronic inflammation and IM progression caused by DCA in INS-GAS mice, and these findings were consistent with the aforementioned gastric epithelial cell and organoid results.

**Figure 9.**
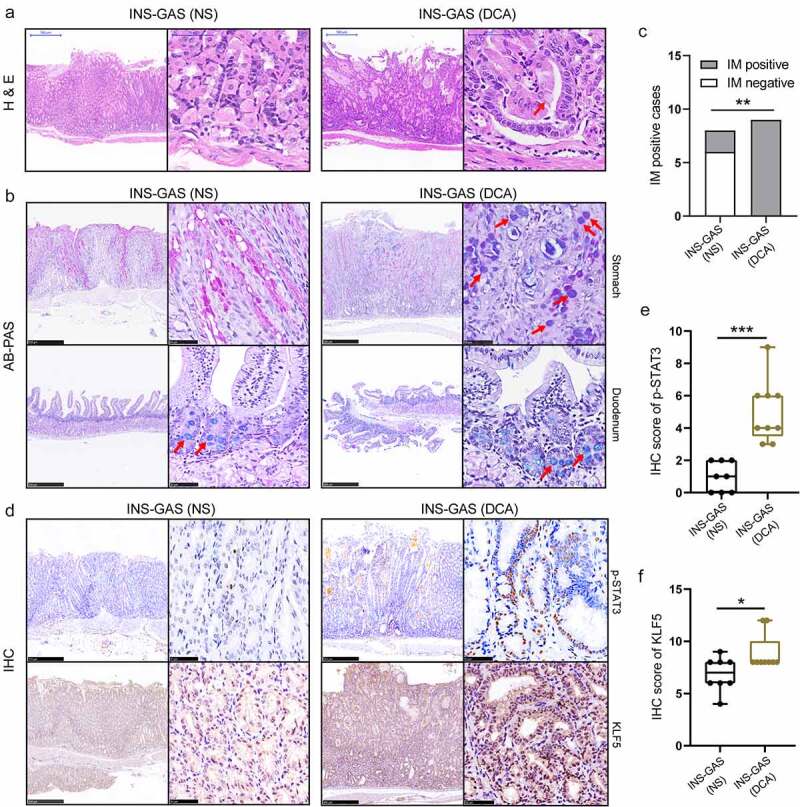
Effects of DCA on gastric carcinogenesis in INS-GAS mice assessed by H&E, AB/PAS and IHC staining. (a) Representative images of H&E staining from the two groups of INS-GAS mice. The red arrow shows a goblet cell in a dysplastic gastric gland. (b) Representative images of AB/PAS staining in the gastric and duodenal mucosae of the two groups. Duodenal sections containing intestinal mucus were stained blue as positive references for reaction with AB. The red arrows indicate AB positive staining. (c) Comparison of the number of gastric IM cases between the two groups (Fisher’s exact test). (d) IHC staining for p-STAT3 and KLF5 in the stomach sections of the two groups. The right boxes depict enlarged regions of the left images. Scale bars represent 500 μm (left) and 50 μm (right). (e-f) IHC staining scores of p-STAT3 and KLF5 between the two groups.

We next histologically assessed the effects of DCA on gastric carcinogenesis in INS-GAS mice by H&E, Alcian blue and periodic acid-Schiff (AB/PAS) and IHC staining. First, H&E staining of the gastric mucosa of INS-GAS mice revealed accelerated gastric IM and low-grade dysplasia (LGD) after DCA treatment. The red arrow in the representative image indicates a typical goblet cell in an irregular gastric gland in the DCA group (Figure 9a). Second, since 65.2% of cases of IM columnar cells that secreted intestinal mucus showed AB-positive staining due to the presence of acidic mucin,^44^ AB/PAS staining was further performed to identify the type of mucin. Consequently, mucosal columnar cells in the control mice were stained magenta with PAS, indicating the presence of neutral mucin, whereas columnar cells in the bottoms of glands and the fovea of gastric mucosa in all cases in the DCA group reacted with AB and were stained blue-violet (Figure 9b). Considering both the presence of goblet cells and/or the presence of acidic mucin, all INS-GAS mice that received DCA treatment developed gastric IM at 6 months (9/9, 100%), while only 2 of 8 (25%) mice in the control group were identified as having gastric IM (*P* < .01; Figure 9c). Furthermore, we tested p-STAT3 and KLF5 expression levels by IHC staining and further scored the corresponding nuclear staining intensities (Figure 9d-f). Compared with those of the control mice, the nuclear staining intensities of p-STAT3 and KLF5 in the gastric tissues of the DCA group were remarkably higher (1.00 ± 0.33 *vs*. 5.00 ± 0.65, *P* < .001; 6.88 ± 0.58 *vs*. 8.89 ± 0.59, *P* < .05). In summary, DCA resulted in the systemic accumulation of TBA and enhanced STAT3 phosphorylation and IM marker expression in the gastric tissues of INS-GAS mice. Next, we explored DCA-mediated alterations in the gastric microenvironment in INS-GAS mice, including changes in gastric BA metabolism and microbial structure.

### DCA induces gastric environmental alterations involving abnormal BA metabolism and microbial dysbiosis

**Figure 10.**
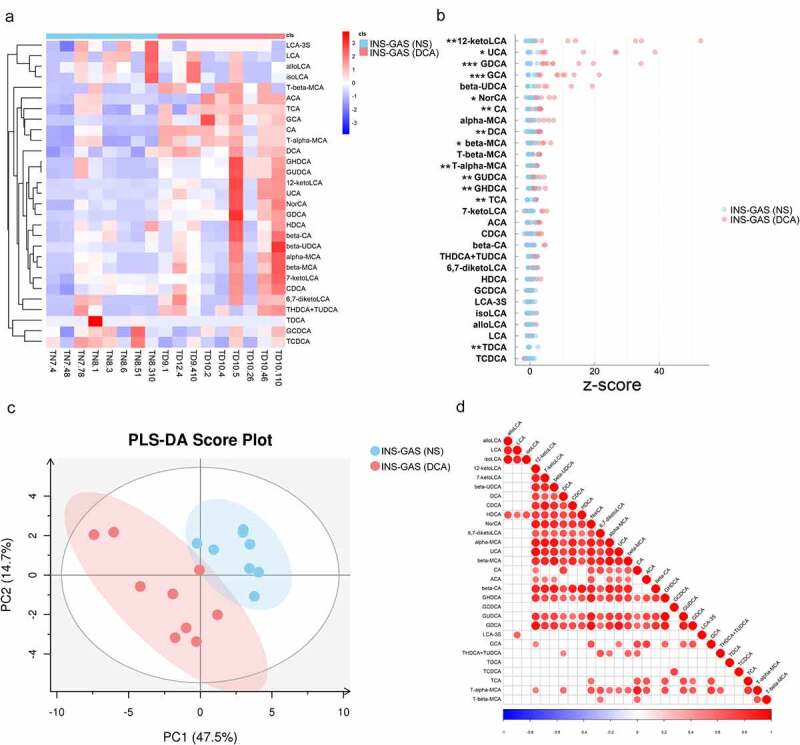
Alterations in gastric BA metabolites in INS-GAS mice treated with DCA detected by a targeted metabolomics approach. (a) Heatmap of the relative concentrations of BAs in the two groups determined by agglomerate hierarchical clustering analyses. (b) Z score plot based on BA concentrations in the stomachs of the two groups. (c) PLS-DA score plot of gastric BA metabolites. PLS-DA model validation parameters: Pre 2, R2X (cum) 0.622, R2Y (cum) 0.834 and Q2 (cum) 0.674. (d) Correlation heatmap of gastric BA metabolites based on Pearson correlation analyses. *P < .05, **P < .01, ***P < .001.

To identify whether BA metabolites other than DCA promote IM occurrence, we performed a BA-targeted metabolomics approach on gastric content samples from DCA-supplemented mice and control mice, with both groups possessing a transgenic INS-GAS background. The gastric BA concentrations in the two groups are shown in Figure 10(a,b), and those with significant differences between the two groups are listed in **Table S1**. The concentrations of twelve BAs (UCA, ursocholic acid; GCA, sodium glycocholate hydrate; 12-ketoLCA, 12-ketolithocholic acid; GDCA, glycodeoxycholic acid; β-MCA, β-muricholic acid; CA, cholic acid; T-α-MCA, tauro-α-muricholic acid; GUDCA, glycoursodeoxycholic acid; NorCA, norcholic acid; GHDCA, glycohyodeoxycholic acid; DCA, deoxycholic acid; and TCA, taurocholic acid) were greater in gastric content samples from the DCA group compared with the controls. A partial least squares-discriminate analysis (PLS-DA) plot showed distinct clustering patterns between the DCA-supplemented mice and control mice (Figure 10C). Additionally, correlation analyses revealed that the concentrations of all BA metabolites were positively correlated except for GCDCA and TDCA (Figure 10D).

**Figure 11.**
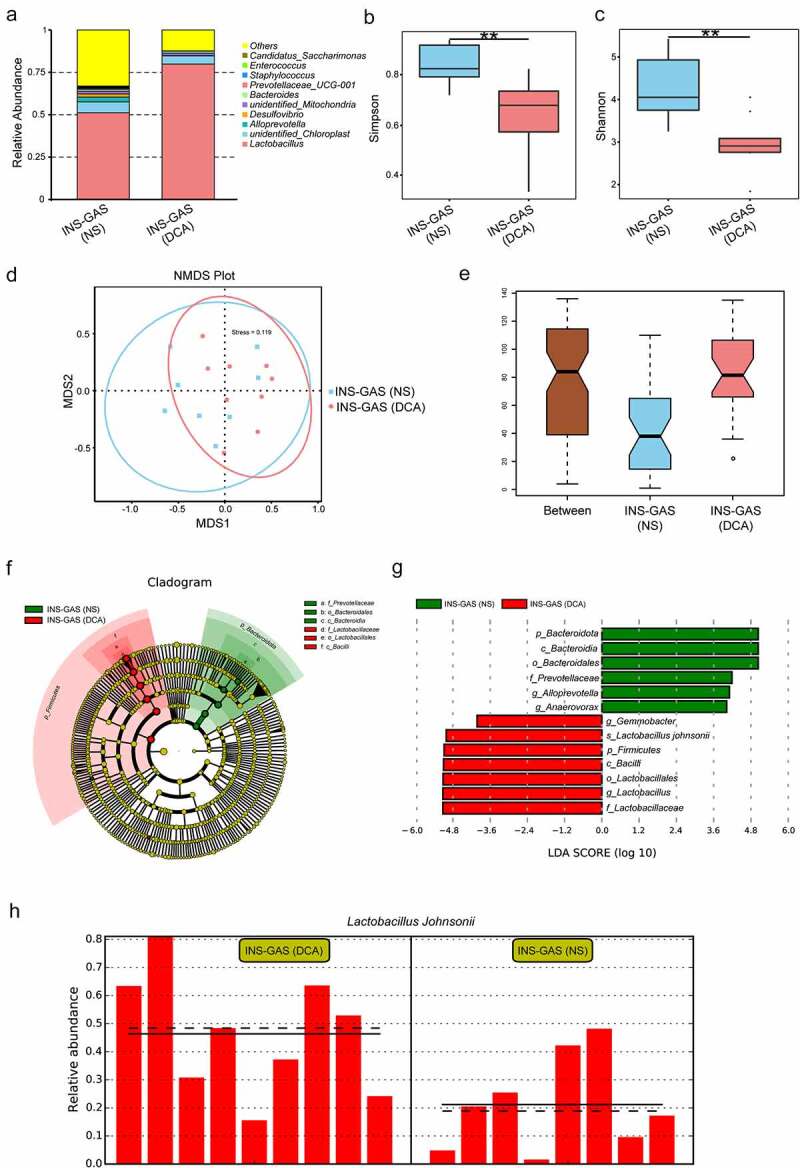
16S rRNA gene sequencing results of the gastric contents of INS-GAS mice treated with DCA. **(A)** Top ten gastric bacterial genera with the highest relative abundance in both groups. **(B** and **C)** Simpson index and Shannon index comparison of the α-diversity of gastric microbes between the two groups. **(D** and **E)** β-diversity comparison between the two groups based on NMDS and ANOSIM analyses. **(F** and **G)** Significantly different taxa enriched in the gastric microbiota of the two groups. **(H)** Comparison of the relative abundance of *Lactobacillus johnsonii* in the two groups.

Deep sequencing of the 16S rRNA genes in the gastric contents was used to determine the microbial profiles in the stomachs of INS-GAS mice treated with DCA and NS. The top three bacterial genera in terms of relative abundance were *Lactobacillus, unidentified_Chloroplast*, and *Alloprevotella* in both groups (Figure 11A). Microbial α-diversity analysis revealed that the microbial richness and evenness in the DCA-treated group was significantly lower than that in the NS control group (Figure 11(B,C), Simpson index and Shannon index, both with *P* < .01). NMDS and ANOSIM analyses were used to compare the β-diversity and showed a significant difference in microbial community structure between the two groups (Figure 11D, Stress = 0.119; Figure 11E, R = 0.2205, *P* = .015). We further screened potential DCA-induced cancer-specific microbial candidates in INS-GAS mice. At the genus level, significantly increased abundances of *Gemmobacter* and *Lactobacillus* and significantly decreased abundances of *Alloprevotella* and *Anaerovorax* were observed in the DCA group compared to the control group (Figure 11 (F,G). Interestingly, at the species level, we found that the relative abundance of *Lactobacillus johnsonii* was significantly higher in the DCA group than in the control group (Figure 11 **G and** H). GC-associated genus *Lactobacillus* was enriched in the stomachs of *H. Pylori*-infected INS-GAS mice, while the beneficial short-chain fatty acids-producing bacteria including *Alloprevotella* were more abundant in *H. Pylori*-infected mice with subsequent probiotics supplementation,^45^ which was consistent with our findings. However, gastric wash samples of patients with GC and superficial gastritis were detected by shotgun metagenomic sequencing, and it was found that the genus *Alloprevotella* that usually colonizes the oral cavity was highly abundant in GC.^46^ Therefore, unlike genus *Lactobacillus*, the pathophysiological roles of genus *Alloprevotella* in the mouse and human stomachs are inconsistent. In summary, DCA mediated decreased bacterial diversity and increased microbial dysbiosis in INS-GAS mice, especially enriching the *Lactobacillus* genus.

### Multiomics analyses showed that *Lactobacillus* genus enrichment was positively correlated with increased levels of GCA, CA, T-α-MCA, TCA and β-MCA in DCA-treated INS-GAS mice

**Figure 12.**
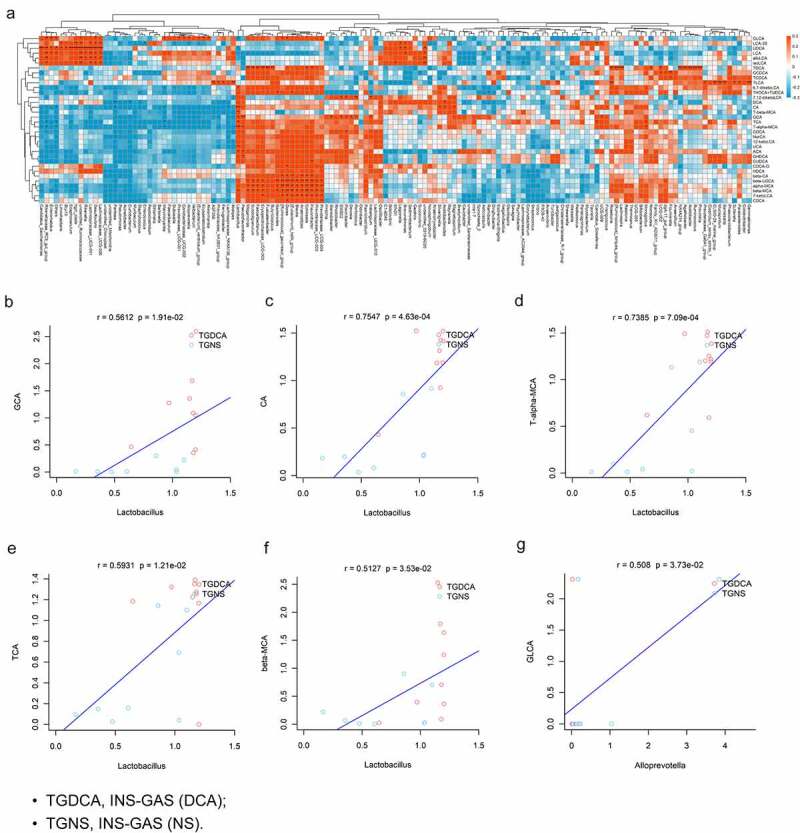
Multiomics analyses showed that *Lactobacillus* genus enrichment was positively correlated with increased levels of GCA, CA, T-α-MCA, TCA and β-MCA in DCA-treated INS-GAS mice. (a) Heatmap of the multiomics correlation analyses of BA profiles and gastric microbes at the genus level. Pearson correlation analyses of the *Lactobacillus* genus and the levels of GCA (b), CA (c), T-α-MCA (d), TCA (e) and β-MCA (f) in the stomachs of INS-GAS mice. (g) Pearson correlation analysis of the *Alloprevotella* genus and the level of GLCA in the stomachs of INS-GAS mice. *P < .05, **P < .01, ***P < .001.

**Figure 13.**
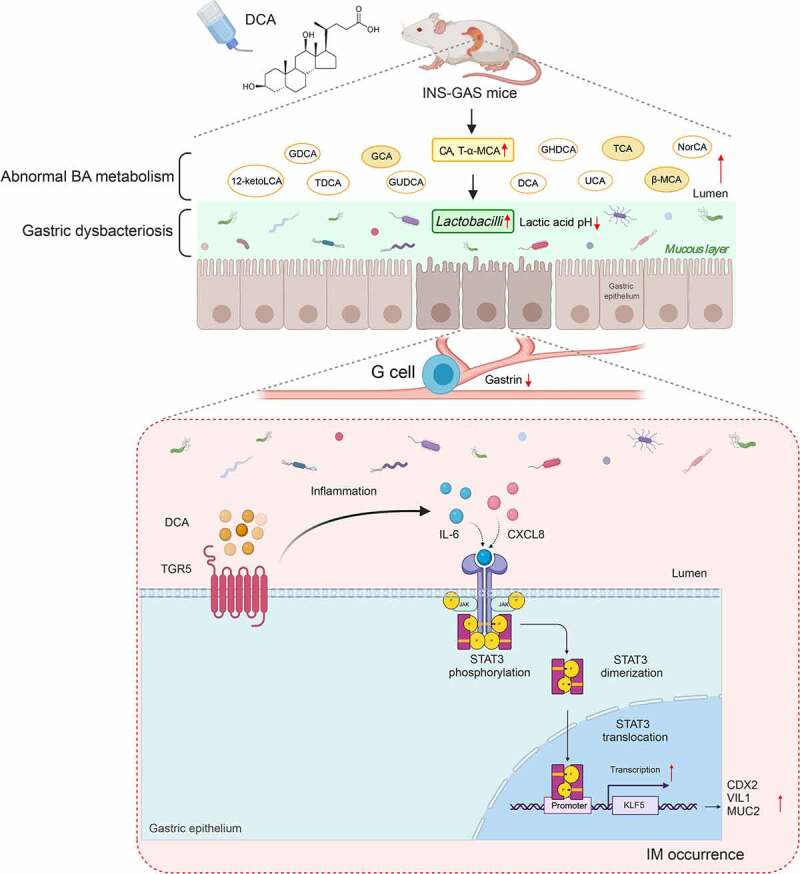
Mechanisms underlying gastric intestinal metaplasia caused by secondary bile acids. DCA activates the TGR5-STAT3-KLF5 axis in gastric tissues and disturbs gastric BA metabolism and microbiota in INS-GAS mice.

Previous studies have depicted bile acid-microbiota crosstalk in the gastrointestinal tract. On the one hand, BAs regulate the microbial structure, while on the other hand, gut microbiota modulate the size and composition of the BA pool, which consequently contributes to digestive diseases and even cancer.^47^ In this study, we explored the interactions between BA profiles and gastric microbes during the process of IM and carcinogenesis in the gastric mucosa caused by DCA in INS-GAS mice. The results of multiomics correlation analyses of the BA profiles and gastric microbes at the genus level are presented in Figure 12A. Since the abundance of the *Lactobacillus* genus and the aforementioned twelve BA species levels were upregulated in the DCA group compared with the controls, we further performed correlation analyses of the *Lactobacillus* genus and the levels of these BA species and revealed that the *Lactobacillus* genus was significantly positively correlated with GCA, CA, T-α-MCA, TCA and β-MCA in the stomachs of INS-GAS mice administrated DCA (Figure 12B-F). Among them, CA and T-α-MCA had the highest correlation with DCA (r = 0.7547 and 0.7385). Furthermore, as shown in Figure 10A, CA and T-α-MCA were clustered closely with each other. More specifically, eleven of the twelve BA species had significant positive correlations with *Lactobacillus johnsonii* enrichment (**Fig. S3A-K**). These data indicate that the *Lactobacillus* genus might be a potential IM microbial marker that interacts with gastric BA metabolites and is involved in BA-induced gastric IM occurrence and development. Besides *Lactobacillus* genus, *Alloprevotella* genus was only significantly positively correlated with GLCA (Figure 12G). There was no significant correlation between *Gemmobacter* and *Anaerovorax* genera and BAs in the stomach. The mechanisms underlying gastric IM caused by DCA are shown in Figure 13. In conclusion, DCA induces gastric IM by activating the TGR5-STAT3-KLF5 axis and disturbing gastric BA metabolism and microbiota.

## Discussion

Chronic DGR, wherein BAs abnormally reflux from the duodenum into the stomach, is another main risk factor in addition to Hp infection for the development of gastric IM and its progression to intestinal-type gastric adenocarcinoma.^11,12^ In this study, we found that exposure to DCA, as the predominant secondary BA in the stomach, activates a novel signaling axis comprising TGR5-STAT3-KLF5 in the gastric epithelium. We also revealed that in addition to altered molecular signaling, altered gastric conditions are also essential for IM development. The long-term increase of the DCA concentration dramatically influenced the metabolism of BAs and dominant bacteria in the stomach. Therefore, bile reflux leads to IM and gastric carcinogenesis in a complex and comprehensive manner, at least evolving abnormal molecular pathways, bile acid metabolism, and microbial structures.

Since dysplastic and subsequent intestinal-type cancerous lesions arise within regions of preexisting IM, the identification of IM markers indicating the risk for the transition from IM to dysplasia is valuable for clinical diagnoses and prognoses. Spasmolytic polypeptide-expressing metaplasia (SPEM) is the earliest metaplasia form derived from atrophy, which then evolves into complete and incomplete IM under chronic inflammation.^48–50^ Although incomplete IM is a major risk factor that ultimately leads to predisposure to adenocarcinoma compared with complete IM, biopsies with H&E staining can only detect a mixture of SPEM lesions and complete and incomplete IM. The INS-GAS mouse model demonstrates the full spectrum of progressive metaplastic lineages from SPEM to IM, dysplasia and finally tumorigenesis.^48^

The interaction between Hp infection and bile reflux in driving gastric IM is complex and controversial. From one perspective, a clinical study showed that premalignant lesions (atrophic gastritis and IM) were more common in patients with both DGR reflux and Hp infection than in those with only DGR reflux (37.4% *vs*. 32.2%), but the association was not statistically significant.^51^ Mechanistically, chronic Hp infection contributes to antroduodenal motility disorder, which indirectly induces the retrograde passage of alkaline duodenal content into the stomach.^51^ Additionally, the presence of DGR does not interfere with the presence and severity of *H. pylori*.^52^ Therefore, together with our findings presented herein, the eradication of Hp without the treatment of DGR might not be sufficient to prevent gastric epithelial carcinogenesis.

STAT3, as a DNA-binding transcription factor, is critical for mediating normal cellular processes upon tyrosine phosphorylation.^53^ The importance of STAT3 signaling in inducing chronic gastritis is now widely accepted. However, the molecular mechanisms underlying how STAT3 signaling drives the transformation of chronic mucosal inflammation to IM have not been clarified. Our study provided evidence that STAT3 was continuously phosphorylated and underwent nuclear accumulation during DCA-induced inflammation and IM and directly bound to the two sites on the promoter of KLF5 to activate its transcription in gastric cells. Furthermore, we also demonstrated that DCA induced the upregulation of other STAT3 target genes, including Mcl-1, Bcl-2, IL-6 and CXCL8; thus, DCA-treated cells showed inflammatory and apoptosis-resistant phenotypes different from those of normal cells. Hence, we present data showing that STAT3 may be a key mediator of carcinogenesis in BA-exposed IM tissues. The secondary BA receptor TGR5 may be the link between BAs and STAT3 activation. Nevertheless, other downstream molecules mediating STAT3-induced IM need to be further verified in future studies.

We found that *Gemmobacter* and *Lactobacillus* were DCA-induced IM-associated genera in the stomachs of INS-GAS mice. In addition to Hp, microbial factors may contribute to the progression of gastric carcinogenesis. Several studies have revealed that *Lactobacillus* widely colonizes the human gastric mucosa.^54,55^ It can metabolize lactose into lactic acid, thus acidifying the gastric mucous layer^56^ and subsequently inhibiting gastrin secretion by antral G cells and gastric acid secretion.^57^ Therefore, a higher relative abundance of the *Lactobacillus* genus in the gastric microbiota might accelerate atrophy, IM and tumorigenesis in the gastric mucosae in INS-GAS mice, which have a natural loss of parietal cells and hypochlorhydria. Moreover, the prevalence of *Lactobacillus* in patient stomachs was found to increase gradually from gastritis to gastric metaplasia and cancer by 16S rRNA gene sequencing analysis.^58–60^ In healthy stomachs, *Lactobacillus* is a commensal bacterial genus with a relative abundance of up to 30%.^55,61^ Noteworthily, a case-control study reported that *Lactobacillus* was the dominant genus in GC patients without Hp infection, and its relative abundance was 35.2%-97.0%.^62^ These results are in accordance with our study, which showed that DCA induced gastric IM and dysplasia along with *Lactobacillus* genus enrichment in INS-GAS mice. However, the mechanism by which DGR leads to increased *Lactobacillus* abundance in the stomach is still poorly understood. Additionally, our study is the first to depict the increased abundance of the *Gemmobacter* genus in the tumorigenic stomachs of INS-GAS mice exposed to DCA for a prolonged period.

To the best of our knowledge, this is the first study that used several gastric organoid lines derived from C57BL/6, FVB/N wild-type and FVB/N INS-GAS mice with DCA treatment, and the results suggest that secondary BAs may be tightly linked to the etiology of IM. Moreover, we first revealed that DCA altered the BA metabolism profile and disrupted microbiome homeostasis in the stomachs of INS-GAS mice, which is a mouse model with complete metaplastic lineages for dysplasia and adenocarcinoma. Notably, these findings remain murine-specific, and the administration of only DCA simplified the BA mixture that regurgitates into the stomach during DGR. In animal studies, DCA had been orally administered to mice in the opposite direction of that occurring in actual DGR. To date, there is no appropriate mouse model available to mimic the pathologic process of duodenal content reflux from the duodenum into the stomach cavity. This study provides the first step in indicating that DCA, as a representative secondary BA in the stomach, mediates IM at least at the levels of molecular epigenetics, BA metabolism and microbiota. In future studies, a more appropriate animal model is needed to mimic DGR.

## Conclusion

In summary, our findings suggest that TGR5-STAT3-KLF5 regulatory signaling is essential for the generation of the IM phenotype in gastric epithelium exposed to BAs. The inhibition of TGR5 may be a preventive approach for gastric IM in patients with bile regurgitation. Phosphorylated STAT3 may be useful in the early detection of gastric tumorigenesis. The expression of KLF5 within IM regions may be an indicator of an increased risk for intestinal-type gastric tumorigenesis in the bile-exposed gastric epithelium. The enrichment of the *Lactobacillus* genus with carcinogenic potential was positively correlated with increased concentrations of specific BAs induced by DCA.

## Materials and methods

### Study population

A total of 161 volunteers without Hp infection or cholecystectomy history were recruited to undergo upper endoscopic examination and provide corpus biopsy samples for histological analysis, immunohistochemical staining and RNA extraction in an epidemiological screening program for GC in rural areas of Yangzhou, China. Hp infection status was examined by the rapid urease test. Those with bile reflux into the gastric cavity due to nausea and vomiting caused by the invasive endoscopic procedure were excluded. Finally, obvious primary bile reflux and bile-stained gastric mucosa were observed in 10 subjects under endoscopic views. Among them, 4 subjects were found to have IM based on mucosal specimens, while the remaining subjects were reviewed as histologically normal based on the Updated Sydney System.^63^ Six volunteers without bile reflux or histopathological IM were randomly selected as the control group for further analysis. General information about the age, sex, bile reflux status and histological assessment of the 16 enrolled subjects is listed in **Table S2**. This study was approved by the ethics review board of Nanjing Medical University (number 2018-SR-285).

### Cell culture and treatment

The normal human gastric epithelial cell line GES-1 and GC cell lines AGS, SGC-7901, BGC-823 and MKN-45 were cultured in RPMI 1640 medium supplemented with 10% fetal bovine serum (FBS) and 1% penicillin-streptomycin solution (Beyotime, Haimen, China) and placed in a humidified incubator containing 5% CO_2_ at 37°C. DCA power was purchased from Sigma-Aldrich (St. Louis, MO, US) and dissolved in DMSO to prepare a DCA stock solution. The DCA stock solution was further diluted in DMEM medium to the required experimental concentration. For the untreated control group (UT), an equal amount of DMSO was diluted in DMEM medium. The purpose of diluting DCA stock solution with DMEM medium is to reduce the amount of DMSO to reduce the effect of DMSO toxicity on cell viability.

Mouse gastric organoid lines extracted from the corpus region of the C57BL/6, FVB/N and INS-GAS mice were established and maintained according to the instructions of IntestiCult™ Organoid Growth Medium (Stemcell Technologies, Vancouver, Canada; Figures 6a and 7a). The cells were resuspended in cold Cultrex PathClear Reduced Growth Factor Basement Membrane Extract (Bio-techne, Minneapolis, USA) and added to wells for incubation at 37°C for 10 min. After polymerization, the cell droplets were overlaid with organoid growth medium to develop into organoids in one week.

### Mouse treatment

All mouse studies were approved by the Animal Core Facility of Nanjing Medical University (January 2020, Approval No. IACUC-2001024). INS-GAS mice aged 2–3 months were treated with 0.2% DCA in their drinking water for 6 months, and this DCA dosage was based on previously published literature.^43^ Meanwhile, transgenic littermates in the control group were fed normal saline (NS) in their drinking water (Figure 8a). The water in both the DCA and NS groups was changed twice a week. The mice were monitored daily and weighed weekly throughout the experiment. After the mice were anesthetized at the end of month 6, approximately 0.6–1 mL of blood was collected from the orbital. Then, the mice were sacrificed, and the whole stomachs were removed. After incision along the greater curvature of the stomach, the gastric tissues were used for histopathological analyses, western blotting and qRT-PCR assays. The gastric contents were quickly frozen in liquid nitrogen until use for BA metabolite measurement and microbiota sequencing.

### RNA extraction and quantitative real-time PCR (qRT-PCR)

Total RNA was isolated from gastric cells and tissues using TRIzol reagent (Invitrogen, USA). Reverse transcription was performed using Super-Script II (TaKaRa, Japan) with 1000 ng of RNA from each sample, and qRT-PCR for mRNA amplification was performed with a SYBR Green Master Mix kit (TaKaRa). The target mRNA level was normalized to the GAPDH level. The PCR primer sequences are listed in **Table S3**.

### Cell fractionation and western blotting

Cell fractionation was performed by using a nuclear and cytoplasmic protein extraction kit (Beyotime Biotechnology, Shanghai, China). Briefly, the cells were scraped off and added to ice-cold cytoplasmic protein extraction buffer A followed by buffer B. The tube was vortexed and then centrifuged at 12000 rpm for 5 min at 4°C. The supernatant cytoplasmic fraction was transferred to a new tube. The remaining pellet was resuspended in ice-cold nuclear protein extraction buffer and then centrifuged at 12000 rpm for 10 min. The supernatant nuclear fraction was also transferred to another new tube. The cytoplasmic and nuclear fractions were subjected to western blotting assays.

Total proteins were obtained from the cell samples, organoid samples and stomach tissues by complete lysis with protease and phosphatase inhibitors. Denatured proteins (15–30 mg) were separated by using an SDS-PAGE system and transferred onto PVDF membranes. The membranes were blocked with 5% bovine serum albumin for 1 h, hybridized with primary antibodies at 4°C overnight and finally immunoblotted with the corresponding HRP-labeled secondary antibodies for 1 h at room temperature. The primary antibodies used were as follows: Stat3 (cat. #9139), phospho-Stat3 (Tyr705, cat. #9145), CDX2 (cat. #12306), cleaved Caspase-3 (cat. #9664), cleaved Caspase-9 (cat. #7237), Mcl-1 (cat. #39224), Bcl-xL (cat. #2764) (all from Cell Signaling Technology, Danvers, USA), GPBAR1 (cat. BS60582) and Lamin B1 (cat. AP6001) (both from Bioworld Technology, Co. Ltd., Nanjing, China), KLF5 (cat. 61099, Active Motif, Carlsbad, USA), and GAPDH (cat. AF5009, Beyotime, Shanghai, China). Blot bands were sprayed with HRP substrate reagent (WBKL0100, Millipore, USA) and visualized with a Bio-Rad ChemiDoc XRS+ imaging system. The band images were quantified using ImageJ software and normalized to the loading control tubulin β or GAPDH. Nuclear fractions were normalized to Lamin B1.

### Enzyme-linked immunosorbent assay (ELISA)

After GES-1 cells were stimulated with 200 μM DCA for 15 min and then incubated in normal medium for 24 hours of recovery, the concentrations of IL-6, CXCL8 and IL-11 in the supernatant were determined with a Human IL-6 ELISA kit (NeoBioscience cat. EHC007), a Human CXCL8 ELISA kit (NeoBioscience cat. EHC008) and a Human IL-11 ELISA kit (NeoBioscience cat. EHC128.48) according to the manufacturer’s instructions.

### Cell counting kit-8 (CCK-8) assay

The viability of GES-1 cells treated with DCA was measured with CCK-8 (Dojindo, Kumamoto, Japan). Briefly, GES-1 cells were seeded into 96-well plates at 3000 cells per well for 24 h and then treated with DCA at different doses (0, 50, 100, 200 and 400 µM) for different times (6, 12, 24 and 48 h). Finally, each well was incubated with 10 μL of CCK-8 reagent at 37°C for another 1 h and then detected for absorbance at 450 nm. All experiments were performed in quintuplicate.

### Colony formation assay

GES-1 cells were seeded at 800 per well into 6-well plates and then starved in serum-free medium overnight. The GES-1 cells were treated with 200 µM DCA for 15 min, followed by incubation in normal medium for two weeks. The cells were fixed with 4% paraformaldehyde and then stained with 0.1% crystal violet. All macroscopic cell colonies were counted for analysis.

### Flow cytometry for apoptosis analysis

After treatment with DCA at 200 µM for 15 min followed by incubation in normal medium for 24 hours of recovery, GES-1 gastric cells were harvested for apoptosis detection according to the kit manufacturer’s instructions. The GES-1 cells were washed, resuspended in 100 μL of 1 × Bind Buffer at a concentration of 1 × 10^6^ cells/mL, and stained with 5 μL of Annexin V-FITC and 5 μL of PI in darkness for 10 min. The apoptosis rate of these cells was measured by flow cytometry and analyzed by FlowJo V10 software (Tree Star, San Francisco, CA, USA). Three independent experiments were performed for apoptosis analysis.

### Immunohistochemical (IHC) staining and evaluation

Tissue sections for IHC were incubated with 3% hydrogen peroxide for 30 min and then blocked with 5% serum for 20 min at room temperature. The slides were incubated overnight at 4°C with the following primary antibodies: TGR5 (Cat. #BS60582, Bioworld Technology, China), p-STAT3 (Cat. #9145, Cell Signaling Technology, USA), and KLF5 (Cat. #61099, Active Motif, USA). Then, the sections were incubated with HRP-labeled secondary antibodies for 30 min at room temperature, visualized with 3,3′-diaminobenzidine and counterstained with hematoxylin. IHC staining was assessed by a gastrointestinal pathologist (H.J.H.) in a blinded manner and scored on a 12-point scale^64^ based on the following two parameters: 1) staining intensity – 0, negative; 1, weak; 2, moderate; and 3, strong and 2) the proportion of positively stained cells – 0, < 5%; 1, 5–25%; 2, 26–50%; 3, 51–75%; and 4, > 75%. The product of the two primary scores provided a final score of 0–12. The final scores from IHC staining were categorized using the following criteria: 0–4, negative expression (-); 5–8, weak positive expression (+); and 9–12, strong positive expression (++).

### Immunofluorescence (IF) staining

Cells were plated onto glass-bottom dishes and allowed to reach ~30%-40% confluence for IF staining. After DCA intervention, the cells were fixed with 4% paraformaldehyde for 15 min and permeabilized with 0.5% Triton X-100 in PBS for 20 min. In particular, for p-STAT3 immunostaining, the cells were incubated with ice-cold 100% methanol for 10 min at −20°C for fixation and permeabilization. The cells were blocked in blocking buffer for 1 h at room temperature and then stained with the indicated primary antibodies against TGR5 (cat. ab72608, 1:100, Abcam), phospho-Stat3 (Tyr705, cat. #9145, 1:100, CST) and KLF5 (cat. 61099, 1:100, Active Motif) at 4°C overnight. The cells were incubated with Alexa Fluor 488-conjugated, FITC-conjugated, or Cy3-conjugated secondary antibodies (FcMACS, Nanjing, China) diluted in 1× PBS for 1 h at room temperature in a humidifier. Nuclei were stained with DAPI (blue). Immunostaining signals were visualized with a Zeiss confocal microscope (LSM 5 LIVE).

### Luciferase reporter assay

To measure STAT3 transcription activity after exposure to DCA, GES-1, AGS and BGC-823 cells were cotransfected with the p-Stat3-Luc reporter vector and β‑galactosidase plasmid (GeneChem, Shanghai, China) accompanied by Lipofectamine 2000 (Invitrogen, Carlsbad, CA, USA) for 24 h, followed by treatment with DCA (200 µM) for 15 min. After another 24 h of recovery in complete media, the cells were collected, and firefly luciferase activities were measured with a dual-luciferase reporter assay kit (Promega, Madison, WI, USA) using a luminometer, with the values normalized to β-galactosidase levels.

### Oligonucleotide transfection

siRNA Oligo was commercially obtained from GenePharma (Shanghai, China). The sequences of si-NC, si-STAT3 (1382), si-STAT3 (1839) and si-STAT3 (398) used are listed in **Table S4**. GES-1 cells were seeded into 6-well plates, allowed to reach 80%-90% confluence and then transfected with si-NC or si-STAT3 accompanied by Lipofectamine 2000 (Invitrogen, Carlsbad, CA, USA) for 24 h.

### Chromatin immunoprecipitation (ChIP)

A total of 1 × 10^8^ GES-1 cells untreated or treated with 200 μM DCA for 15 min followed by 24 h of recovery, were crosslinked with 1% formaldehyde and quenched by glycine addition. The cell lysates were sonicated to obtain DNA fragments of approximately 100–500 bp. Chromatin was immunoprecipitated with antibodies against phospho-STAT3 (Tyr705, cat. #9145, Cell Signaling Technology) or IgG (cat. #GB111739, Servicebio, China) as a control. DNA-protein immunocomplexes were collected using Protein A/G Agarose beads (Millipore) on a rotator at 4°C for 2 h. After washing, eluting and crosslinking reversal with proteinase K, the IP and input DNA were purified and subjected to qPCR. The primer sequences of the negative control (ChIP NC) were as follows: sense primer, 5’-ACCAAAGGTGCGTGCCA-3’, and anti-sense primer, 5’-TCATGTTGAATTGTAGCTCCCATA-3’. The other two primers designed for pSTAT3-ChIP (ChIP 1 and 2) to enrich the promoter region of the *KLF5* gene are listed in Figure 5e.

### Measurement of serum total bile acids (TBA)

Mouse blood was placed at 4°C overnight and then centrifuged at 3000 rpm for 10 min. The serum TBA concentration was detected by analyzing 100 μL of the upper clear liquid with an enzymatic cycling kit (DiaSys Diagnostics Systems GmbH, Germany) following the manufacturer’s instructions.

### Gastric BA composition analysis using LC-MS

BA concentrations were quantified using ultra-performance liquid chromatography coupled with triple quadrupole mass spectrometry (UPLC-TQMS). One milliliter of methanol was added to 10 mg of gastric content samples and mixed by vortexing and sonicating. The samples were centrifuged at 12,000 rpm at 4°C for 10 min, and then all supernatants were diluted 50 times with methanol. The diluted supernatant was filtered through a 0.22 µm membrane for further analysis. For LC separation, a Waters ACQUITY UPLC® BEH C_18_ column (2.1 mm × 100 mm, 1.7 μm) was used with a flow rate of 0.25 mL/min at 40°C. The injection volume was 5 µL. The mobile-phase solvents were 0.01% formic acid in water (A) and acetonitrile (B). The gradient elution conditions were set at 25% B for 4 min (min 0–4), linearly increased to 30% B during the next 5 min (min 4–9), increased to 36% B for min 9–14, increased to 38% B for min 14–18, increased to 50% B for min 18–24, increased to 75% B for min 24–32, increased to 100% B for min 32–35, and finally linearly decreased to 25% B for min 35–38. The electrospray ionization conditions for MS were set as follows: negative ion source temperature, 500°C; ion source voltage, −4500 V; collision gas, 6 psi; air curtain gas, 30 psi; atomizing gas, 50 psi; and auxiliary gas, 50 psi. Multiple reaction monitoring was used for the MS scans.

### 16S rRNA gene amplicon sequencing

DNA extraction and 16S rRNA gene sequencing of gastric content specimens were performed by BioNovoGene Co., Ltd. (Suzhou, China). The microbial 16S rRNA was amplified using primers 341 F 5′-CCTAYGGGRBGCASCAG-3′ and 806 R 5′- GGACTACNNGGGTATCTAAT-3′ targeting the V3-V4 hypervariable regions. The PCR amplification products were purified using a QIAquick Gel Extraction Kit (Qiagen), and the resulting library was constructed using a TruSeq® DNA PCR-Free Sample Preparation Kit (Illumina) and then sequenced on an Illumina NovaSeq 6000 platform.

Raw tags were trimmed and filtered into effective tags, which were further clustered into operational taxonomic units (OTUs) at 97% similarity using Uparse v7.0.1001 software (http://www.drive5.com/uparse/).^65^ OTU sequences were matched to the SILVA v138 SSUrRNA database (http://www.arb-silva.de/).^66^ Microbial α-diversity was profiled with Simpson and Shannon indices. Microbial community structures (β-diversity) were compared using the weighted UniFrac distance and visualized using a nonmetric multidimensional scaling (NMDS) plot and an Anosim boxplot. The online tool linear discriminant analysis effect size (LEfSe)^67^ was used for taxonomic discovery analysis for microbial biomarker discovery. Multiomics correlation analyses were performed using Pearson correlation analyses, which calculated the correlation between the relative abundance of bacterial taxa at genus levels and BA concentrations.

### Statistical analysis

Statistical analyses were performed by using IBM SPSS Statistics 23.0 and GraphPad Prism 8.0 software. Student’s *t* test or the chi-square test was applied to determine significant differences between two groups, while ANOVA with Tukey’s test was used to analyze significant differences among multiple groups. Pearson’s test was performed to evaluate correlations between two groups. The cell experiments were performed in at least triplicate, and normally distributed data are presented as the mean ± SEM of at least three independent experiments. A 2-sided *P* < .05 was considered significant. The amplicon sequencing statistical analyses were performed with R software. Microbial α-diversity indices were calculated using the Wilcoxon test, while microbial β-diversity was compared between two groups using the ANOSIM nonparametric test.

## Supplementary Material

Supplemental Material

## Data Availability

All data generated or analyzed during this study are included in this published article and its supplementary information files.

## List of abbreviations

AB/PAS, Alcian blue and periodic acid-Schiff; BA, bile acid; Bcl-2, BCL2 apoptosis regulator; Bcl-XL, BCL2 Like 1; BR, bile reflux; CCK-8, cell counting kit-8; CDX2, caudal type homeobox 2; ChIP, chromatin immunoprecipitation; CXCL8, C-X-C motif chemokine ligand 8; DCA, deoxycholic acid; DGR, duodenogastric reflux; ELISA, enzyme-linked immunosorbent assay; FBS, fetal bovine serum; GC, gastric cancer; GIN, gastric intraepithelial neoplasia; GLCA, Glycolithocholic acid; GPBAR1 (also called TGR5), G protein-coupled bile acid receptor 1; Hp, *Helicobacter pylori*; IF, immunofluorescence; IHC, immunochemistry; IL-6, interleukin-6; IL-11, interleukin 11; IM, intestinal metaplasia; INS-GAS, insulin-gastrin; KLF5, kruppel-like factor 5; LGD, low-grade dysplasia; MCL 1, MCL1 apoptosis regulator; MUC2, mucin 2; NFKB1, nuclear factor kappa B subunit 1; NS, normal saline; qRT-PCR, quantitative real-time PCR; STAT3, Signal transducer and activator of transcription 3; TBA, total bile acid; TGR5, also called GPBAR1, G protein-coupled bile acid receptor 1; UPLC-TQMS, ultra-performance liquid chromatography coupled with triple quadrupole mass spectrometry; UT, untreated; VIL1, Villin 1.
