# Supplementary figures and images for: Deoxycholic acid induces gastric intestinal metaplasia by activating STAT3 signaling and disturbing gastric bile acids metabolism and microbiota
Source: Gut Microbes. 2022 Sep 6;14(1):2120744. doi: 10.1080/19490976.2022.2120744 (PMC9467587; doi:10.1080/19490976.2022.2120744)

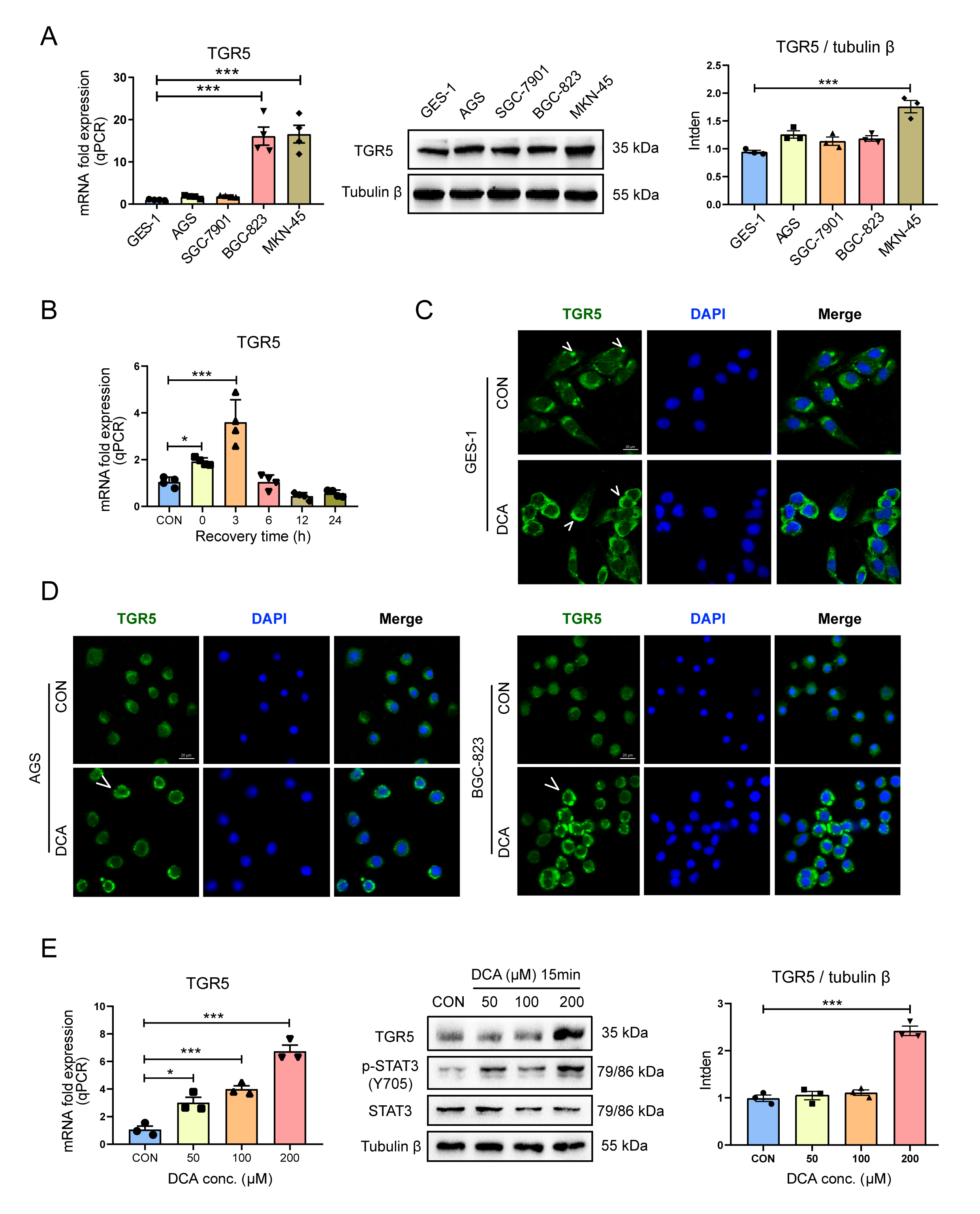

Supplement: Supplemental Material [file KGMI_A_2120744_SM5007.zip › Figure S1 (3).tif]

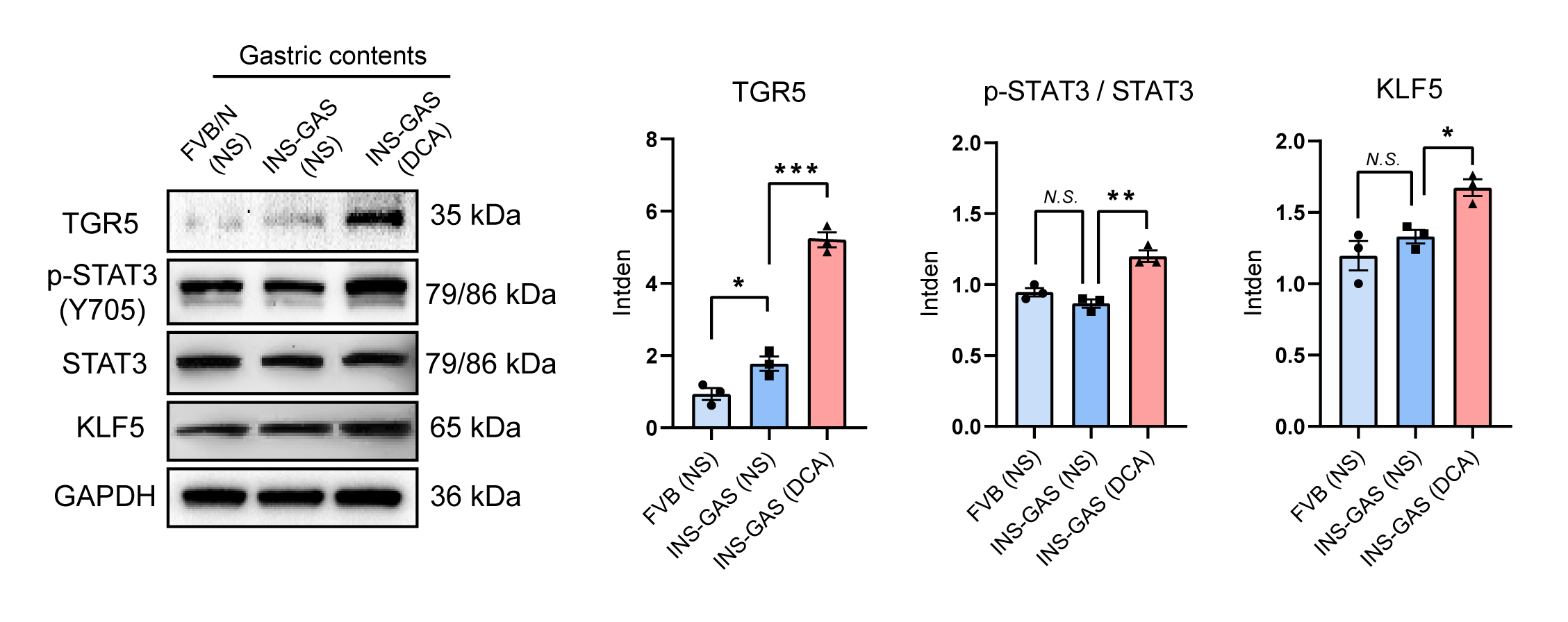

Supplement: Supplemental Material [file KGMI_A_2120744_SM5007.zip › Figure S2 (3).tif]

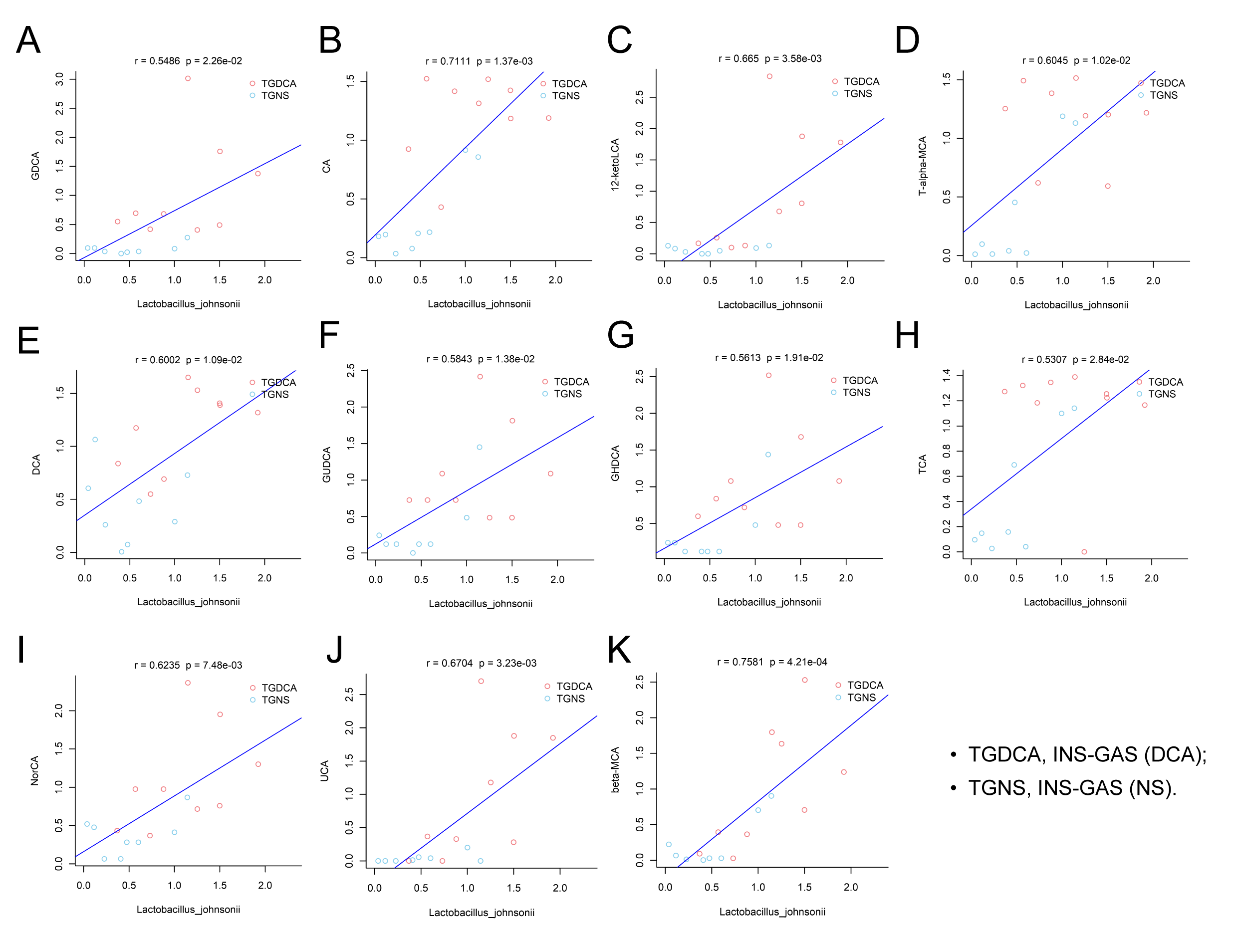

Supplement: Supplemental Material [file KGMI_A_2120744_SM5007.zip › Figure S3 (3).tif]

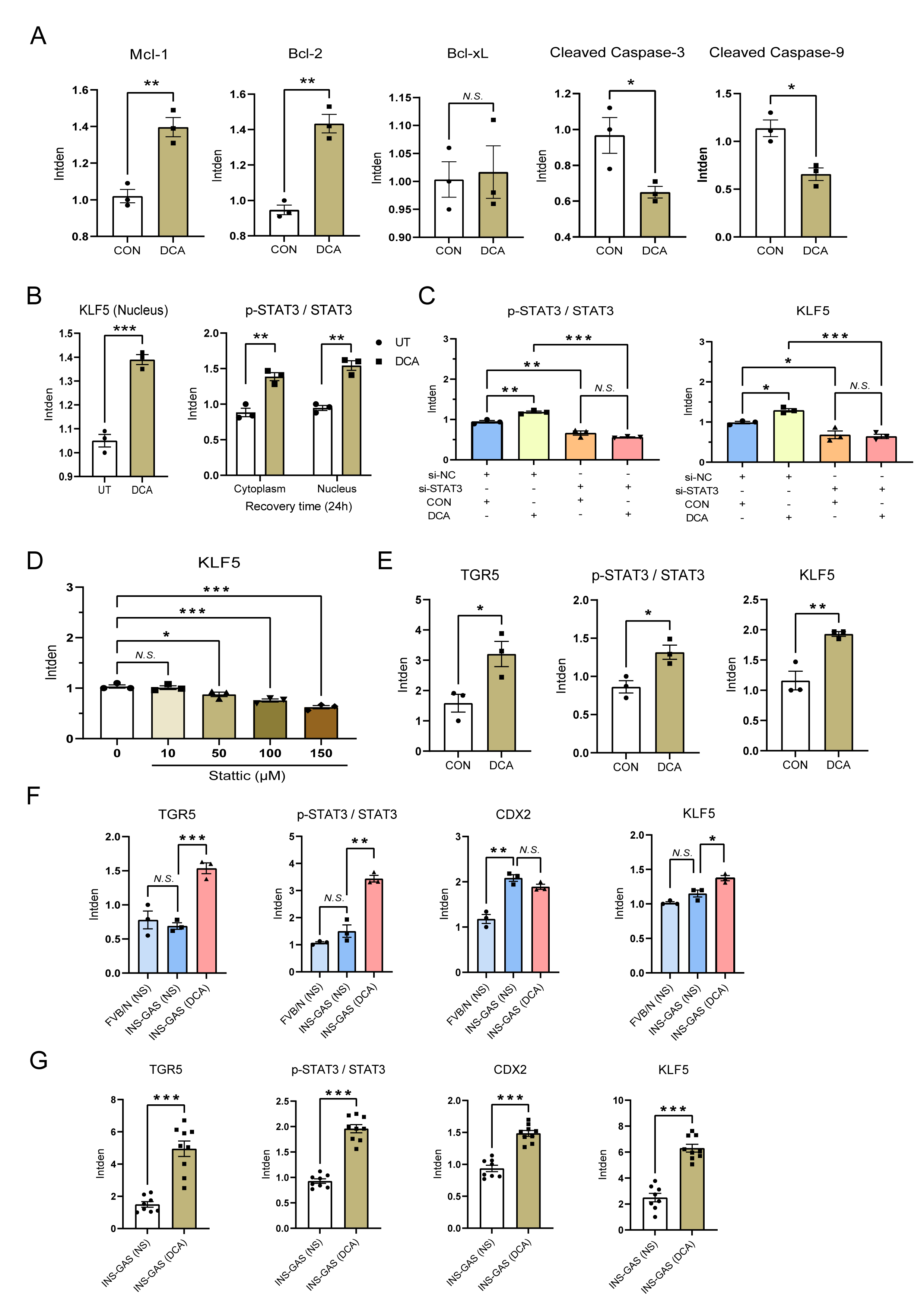

Supplement: Supplemental Material [file KGMI_A_2120744_SM5007.zip › Figure S4 (2).tif]
